# Prdm15 acts upstream of Wnt4 signaling in anterior neural development of *Xenopus laevis*

**DOI:** 10.3389/fcell.2024.1316048

**Published:** 2024-02-20

**Authors:** Ernestine Saumweber, Slim Mzoughi, Arin Khadra, Anja Werberger, Sven Schumann, Ernesto Guccione, Michael J. Schmeisser, Susanne J. Kühl

**Affiliations:** ^1^ Institute of Biochemistry and Molecular Biology, Ulm University, Ulm, Germany; ^2^ Center of OncoGenomics and Innovative Therapeutics (COGIT), Department of Oncological Sciences, Tisch Cancer Institute, Icahn School of Medicine at Mount Sinai, New-York, NY, United States; ^3^ Institute of Anatomy, University Medical Center of the Johannes Gutenberg-University Mainz, Mainz, Germany; ^4^ Focus Program Translational Neurosciences, University Medical Center of the Johannes Gutenberg-University Mainz, Mainz, Germany

**Keywords:** Prdm15, GAMOS, HPE, disease modeling, *Xenopus laevis*, Wnt signaling

## Abstract

Mutations in *PRDM15* lead to a syndromic form of holoprosencephaly (HPE) known as the Galloway–Mowat syndrome (GAMOS). While a connection between PRDM15, a zinc finger transcription factor, and WNT/PCP signaling has been established, there is a critical need to delve deeper into their contributions to early development and GAMOS pathogenesis. We used the South African clawed frog *Xenopus laevis* as the vertebrate model organism and observed that *prdm15* was enriched in the tissues and organs affected in GAMOS. Furthermore, we generated a morpholino oligonucleotide–mediated *prdm15* knockdown model showing that the depletion of Prdm15 leads to abnormal eye, head, and brain development, effectively recapitulating the anterior neural features in GAMOS. An analysis of the underlying molecular basis revealed a reduced expression of key genes associated with eye, head, and brain development. Notably, this reduction could be rescued by the introduction of *wnt4* RNA, particularly during the induction of the respective tissues. Mechanistically, our data demonstrate that Prdm15 acts upstream of both canonical and non-canonical Wnt4 signaling during anterior neural development. Our findings describe severe ocular and anterior neural abnormalities upon Prdm15 depletion and elucidate the role of Prdm15 in canonical and non-canonical Wnt4 signaling.

## 1 Introduction

Congenital disorders can be defined as structural or functional anomalies that manifest at birth as a result of abnormal fetal development, with approximately 3% of births being affected, and they can cause early mortality of newborn infants or lifelong disabilities (Rynn et al., 2008; Swanson and Sinkin, 2013). Holoprosencephaly (HPE) is one of the most common congenital forebrain disorders and is characterized by a wide spectrum of structural anomalies of the brain and midface (Tekendo-Ngongang et al., 1993; Dubourg et al., 2007). Furthermore, clinical features are variable and range from microcephaly and hypertelorism to defects of the eye that include anophthalmia, microphthalmia, and coloboma (Tekendo-Ngongang et al., 1993; Roessler and Muenke, 1998; Ohuchi et al., 2019). In most of the cases, the underlying cause of HPE remains unclear due to the high genetic and clinical heterogeneity and the multi-factorial etiology (Roessler and Muenke, 2010; Roessler et al., 2018). Research over the past decades has uncovered that failure in key signaling pathways during early embryogenesis leads to patterning defects of the forebrain and contributes to cause HPE. Conceivably, any gene involved in patterning of the forebrain could induce HPE, yet only a small set of genes have been functionally investigated and identified to be disease causative (Wallis and Muenke, 2000; Petryk et al., 2015; Roessler et al., 2018; Grinblat and Lipinski, 2019; Mzoughi et al., 2020). HPE may occur isolated (non-syndromic), more likely having a monogenic cause, or as part of a syndrome with chromosomal anomalies or single-gene disorders (Kruszka and Muenke, 2018; Malta et al., 2023).

Mutations in *PRDM15*, which codes for PRDF1 (positive regulatory domain I–binding factor 1) and RIZ1 (retinoblastoma protein–interacting zinc finger gene 1) homology domain–containing protein 15 (PRDM15), result in a syndromic form of HPE also known as the Galloway–Mowat syndrome (GAMOS) (Mzoughi et al., 2020; Mann et al., 2021). To date, 10 different genes have been identified as disease causing for GAMOS, whereby most patients have a mutation in one of the components of the human endopeptidase-like, kinase, chromatin-associated/kinase, endopeptidase-like and other small proteins of small size (EKC/KEOPS) complex (Braun et al., 2017). The main clinical manifestations of this complex congenital syndrome are early-onset steroid-resistant nephrotic syndrome (SRNS) and brain anomalies (Galloway and Mowat, 1968; Ekstrand et al., 2012). GAMOS is often accompanied by abnormal retinal function, facial dysmorphism, and skeletal anomalies (Al-Rakan et al., 2018; Lin et al., 2018; Racine and Golden, 2021). Interestingly, the neural features of GAMOS and HPE share similar clinical features.

Recently, we have identified mutations in *PRDM15* that can cause typical GAMOS renal malformations in embryos of the South African clawed frog *Xenopus laevis* (*X. laevis*) (Mann et al., 2021) and demonstrated that genetic deletion of *Prdm15* in mice recapitulates brain malformations observed in syndromic HPE (GAMOS) patients (Mzoughi et al., 2020). Furthermore, functional studies in mouse embryonic stem cells showed that PRDM15 acts upstream of wingless-type MMTV integration site family member (WNT) and mitogen-activated protein kinase (MAPK) signaling via transcriptional regulation of important genes in both pathways, namely, *Rspo1 (R-spondin-1)* and *Spry1 (sprouty-1)* (Mzoughi et al., 2017). Interestingly, PRDM15 also regulates transcriptional programs of NOTCH and WNT/PCP signaling during anterior axial mesendoderm (AME) specification and anterior/posterior (A/P) patterning of the neural plate to orchestrate forebrain development. Consequently, Prdm15 deficiency leads to severe maldevelopment of the forebrain in mouse embryo (Mzoughi et al., 2020).

Since proper regulation of WNT signaling is fundamental for anterior neural development (Cavodeassi, 2014; Ji et al., 2019; Polevoy et al., 2019), it is crucial to unravel the underlying role of Prdm15 in Wnt signaling during embryogenesis and in congenital diseases.

Here, we observed that *prdm15* is expressed in tissues and organs of the anterior neural tissue affected in GAMOS patients. Furthermore, we generated a morpholino oligonucleotide (MO) *prdm15* knockdown (KD) model in *X. laevis*, revealing that the depletion of Prdm15 caused abnormal eye, brain, and head development. The eye and head phenotypes were significantly rescued by the co-injection of human *PRDM15* wild-type (WT) RNA. Furthermore, we functionally evaluated a previously reported pathogenic human *PRDM15* variant (c.2531G>A, p.C844Y) identified in affected individuals with defective anterior structures and a non-pathogenic variant (c.461T>A; p.M154K) in anterior neural tissue (Mzoughi et al., 2020; Mann et al., 2021). Comparing the rescue of the eye phenotype with the corresponding *hPRDM15* RNA versions, the zinc finger domain variant (C844Y) could only partially rescue the eye phenotype supporting the pathogenic role in the anterior neural tissue of *X. laevis* and in the pathogenesis of GAMOS. Furthermore, we demonstrated that Prdm15 KD leads to a reduced expression of several important genes for proper anterior neural development that could be significantly rescued by *wnt4* RNA co-injection. In addition, Prdm15 KD results in a decreased expression of genes of the Wnt4 signaling pathway such as *wnt4* and its direct target gene *alcam* (*activated leukocyte cell adhesion molecule*). Mechanistically, we uncovered that Prdm15 acts on both the canonical and non-canonical Wnt4 signaling pathways during *X. laevis* embryogenesis.

## 2 Results

### 2.1 Prdm15 is evolutionarily highly conserved and specifically expressed in the anterior neural tissue during *X. laevis* development

Comparative synteny analysis revealed that the location of *prdm15* and its surrounding genes on the chromosome are highly conserved across species (Supplementary Figure S1A). A schematic overview of PRDM15 illustrates the mutations in the PR/SET (c.461T>A, p.M154K and c.568G>A, p.G190K) and zinc finger domains (c.2531G>A, p.C844Y) in GAMOS patients (Mzoughi et al., 2020; Mann et al., 2021) (Figure 3A and Supplementary Figure S1B, C). Interestingly, the full-length protein of human PRDM15 demonstrated similar amino acid lengths and a high evolutionary conservation of the protein sequences across the different species (Supplementary Figure S1D, E). In particular, GAMOS-causing mutations in patients were found in highly conserved domains of the PRDM15 protein sequence (Supplementary Figure S1D, E).

**FIGURE 1:**
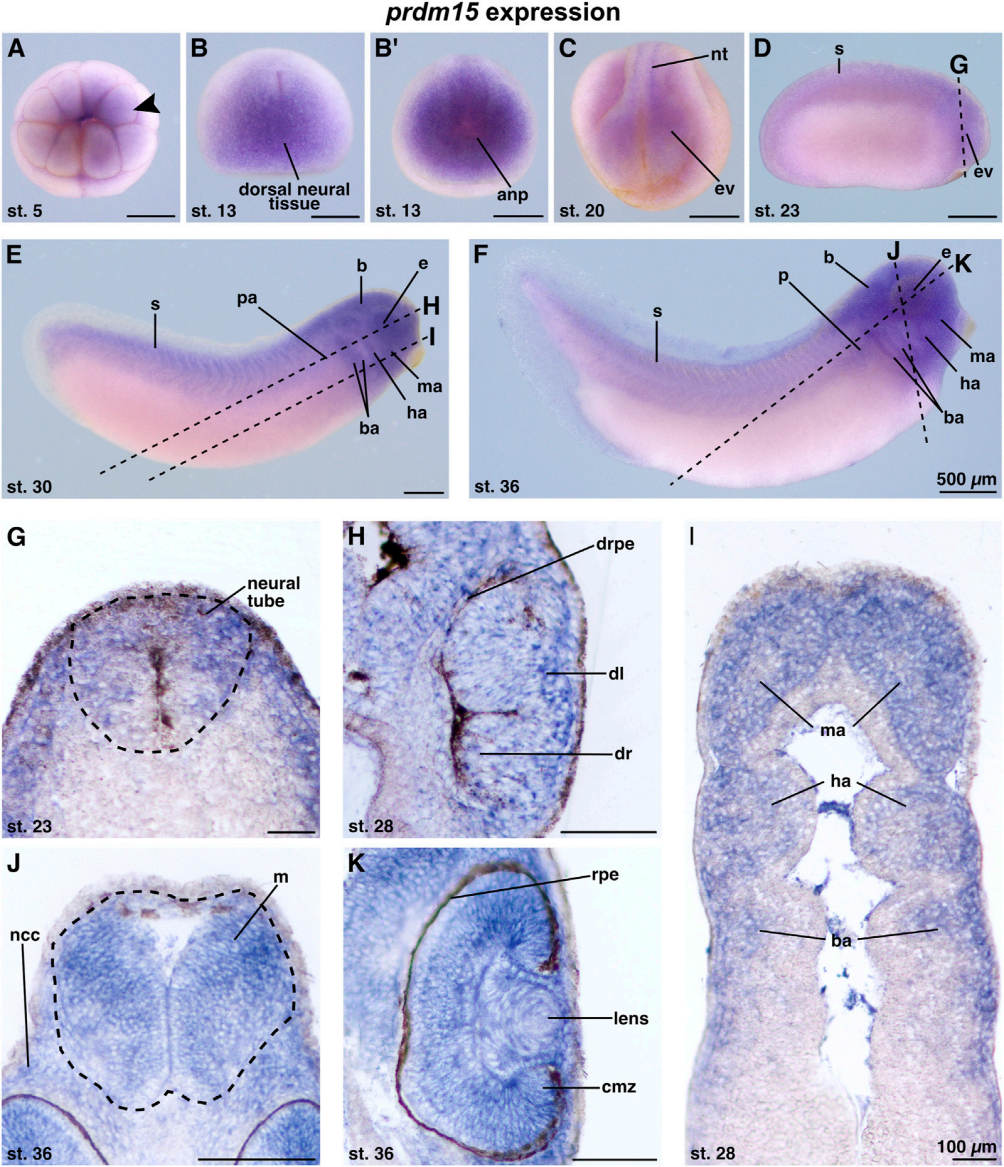
*Prdm15* is expressed during *X. laevis* development in disease relevant tissues of GAMOS. Spatiotemporal expression pattern of *prdm15* visualized by whole-mount *in situ* hybridization (WMISH). Embryonic stages and scale bars are indicated in each panel. Upper part: WMISH with exterior view. Scale bars are equivalent to 500 µm. Black dashed lines represent section planes. Lower part: WMISH following sections. Scale bars are equivalent to 100 µm. **(A)** Vegetal view of *X. laevis* embryos at stage 5 shows a *prdm15* expression in the animal pole (black arrowhead). At stage 13, *prdm15* is strongly expressed in the dorsal neural tissue (**B**, dorsal view) and the anterior neural plate (anp) (**B′**, anterior view). **(C)** At stage 20, *prdm15* is expressed in the neural tube (nt) and in the eye vesicle (ev). **(D)** The lateral view at stage 23 shows *prdm15* transcripts in the somites (s) and the developing eye, more precisely in the eye vesicle (ev). **(E, F)** *Prdm15* expression is detected in lateral views from stages 28 to 36 in the somites (s), the eye (e), the embryonic kidney [pronephric anlage (pa), pronephros (p)], the brain **(B)**, and in the mandibular (ma), hyoid (ha), and branchial arches (ba). **(G)** The transversal section shows an expression of *prdm15* in the neural tube (nt). The horizontal sections **(H, I)** reveal *prdm15* transcripts in the developing retinal pigmented epithelium (rpe), developing lens, and the mandibular (ma), hyoid (ha), and branchial arches (ba). **(J)** The transversal section shows *prdm15* expression in the mesencephalon (m) and neural crest cells (ncc). **(K)** In later stages, *prdm15* is expressed in the eye, more detailed in the lens and ciliary marginal zone (cmz) by horizontal sectioning. Abbreviations: anp, anterior neural plate; b, brain; ba, branchial arch; cmz, ciliary marginal zone; dl, developing lens; dr, developing retina: drpe, developing retinal pigmented epithelium; e, eye; ev, eye vesicle; ha, hyoid arch; m, mesencephalon; ma, mandibular arch; µm, micrometer; ncc, neural crest cells; nt, neural tube; p, pronephros; pa, pronephric anlage; *prdm15*, PR-domain zinc finger protein 15; rpe, retinal pigmented epithelium; s, somite; st., stage; WMISH, whole-mount *in situ* hybridization.

The knowledge of the spatiotemporal expression pattern of *prdm15* during the early development of *X. laevis* remains limited. To date, there is only one other study in *X. laevis* that has described the expression of *prdm15* in the developing eye, branchial arches, and brain of *X. laevis* during two stages in organogenesis (Eguchi et al., 2015). Recently, we have also validated our antisense *digoxigenin*-labeled *prdm15* RNA as suitable for whole-mount *in situ* hybridization (WMISH) and showed a specific expression of *prdm15* in the embryonic kidney of *X. laevis* (Mann et al., 2021). To provide a more detailed expression pattern analysis of *prdm15* during the anterior neural development of *X. laevis*, we used the WMISH technique. At early cleavage stages, *prdm15* transcripts were detected at the animal pole of *X. laevis* embryos (Figure 1A; arrowhead). At stage 13, *prdm15*-positive cells were found in the dorsal neural tissue and the anterior neural plate (Figures 1B, B′). At stage 20, *prdm15* is expressed in the neural tube and eye vesicle (Figure 1C). From stage 23 onward, we found *prdm15* transcripts predominantly in the developing eye, somites, pronephric anlage, brain, and migrating neural crest cells (NCCs) of the mandibular, hyoid, and branchial arches (Figures 1D–F). For detailed analysis, we further performed sections of stained embryos using a vibrating blade microtome. Sections confirmed that *prdm15* transcripts are located in the neural tube and developing eye, specifically in the developing retina, retinal pigment epithelium, and lens (Figures 1G, H). At later stages, *prdm15* is expressed in the mesencephalon, lens, retina, and ciliary marginal zone (Figures 1J, K). Moreover, sections verified an enriched expression in the NCCs that are also located in the mandibular (ma), hyoid (ha), and branchial arches (ba), and next to the mesencephalon (Figures 1I, J).

### 2.2 Prdm15 knockdown results in a severe eye phenotype in *X. laevis*

Our data indicated that during early *X. laevis* development, *prdm15* transcripts were enriched in tissues and organs affected in GAMOS, particularly in the developing eye, NCCs, and brain (Figure 1). We thus used an antisense-based MO KD model to investigate the effects of Prdm15 depletion in the anterior neural development of *X. laevis*. We injected Prdm15 MO unilaterally into one dorsal animal blastomere of *X. laevis* embryos at the eight-cell stage that gives rise to the anterior neural tissue (Moody and Kline, 1990). To monitor the injection efficiency, we co-injected 0.5 ng *GFP* RNA and sorted the embryos according to its specific expression in the neural tissue. A control morpholino oligonucleotide (CoMO) that cannot bind to any *X. laevis* mRNA was used to exclude effects induced by merely injecting liquids and MO (Eisen and Smith, 2008).

**FIGURE 2:**
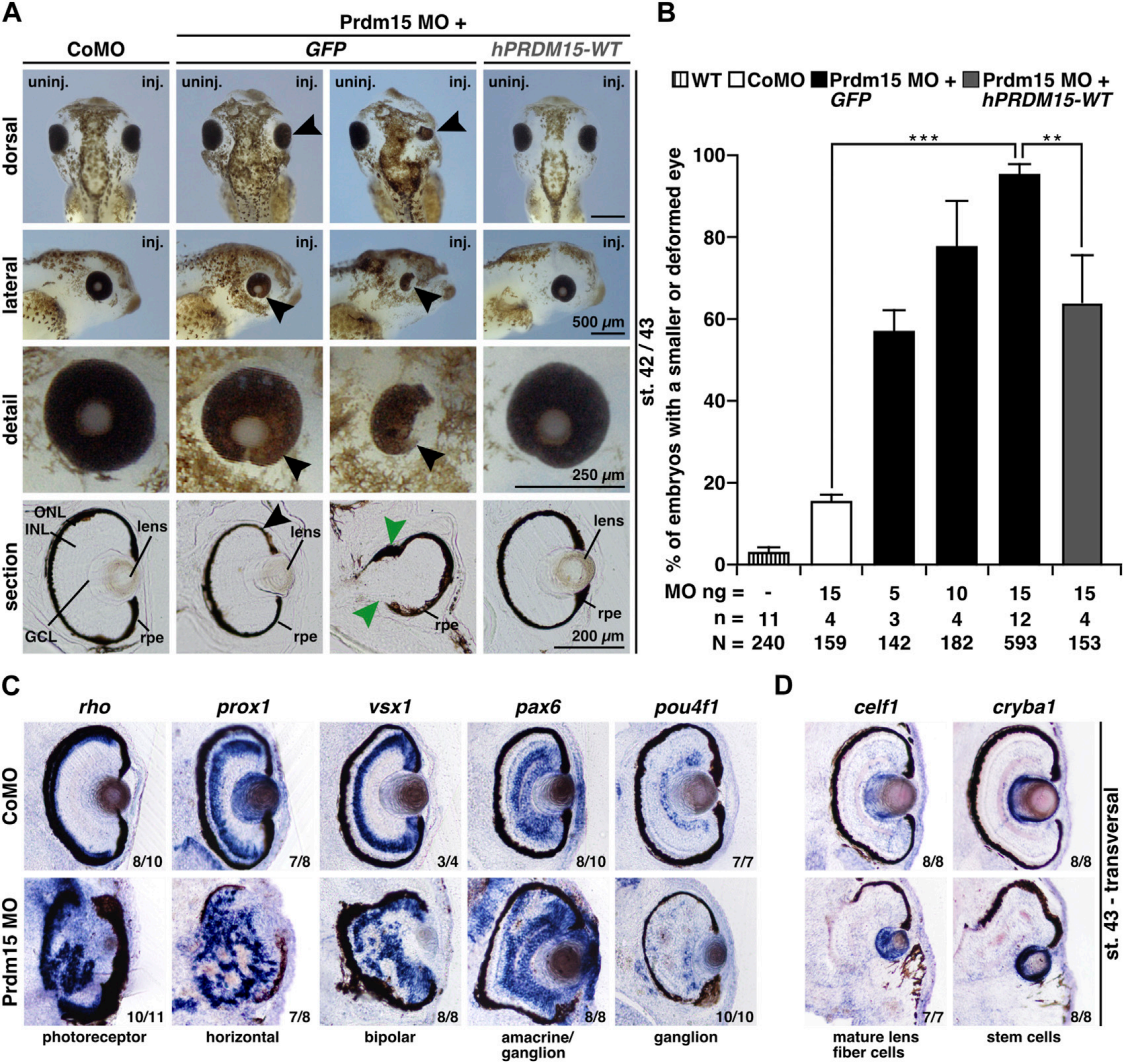
Prdm15 knockdown leads to a severe eye phenotype that is rescued by human *PRDM15* wild-type (WT) RNA. **(A)** Unilateral knockdown (KD) of Prdm15 results in microphthalmia compared to control MO (CoMO), while co-injection of human full-length *PRDM15-WT* RNA rescues the eye phenotype during *X. laevis* eye development. The lateral and detailed views of the embryo show the eye in more detail and the sections specify the lamination of the eye. Black arrowheads point to smaller and deformed eyes, and green arrowheads point to the disturbed and thickened retinal pigmented epithelium (rpe). Representative embryos are shown. **(B)** Statistical evaluation of smaller and deformed eyes as indicated in **(A)**. **(C)** Transversal vibratome sections after whole-mount *in situ* hybridization (WMSH) of Prdm15 MO–injected embryos show a severe eye phenotype in contrast to CoMO-injected embryos. Specific marker genes for retina cell layers are used for specific cell populations of the retina as described in the main text. Most of the cell types are disorganized and displaced. **(D)** Lens-specific marker genes *celf1* and *cryba1* are also affected, showing a smaller expression size, but the organization of the cells is unaffected upon Prdm15 KD. Abbreviations: *celf1*, *CUGBP elav-like family member 1*; CoMO, control morpholino oligonucleotide; *cryba1*, *crystallin beta A1*; GCL, ganglion cell layer; *GFP*, green fluorescent protein; *hPRDM15*, human *PRDM15*; INL, inner nuclear cell layer; inj., injected; MO, morpholino oligonucleotide; n, number of independent experiments; N, number of analyzed embryos in total; ng, nanogram; ONL, outer nuclear cell layer; *pax6*, *paired box 6*; *pou4f1*, *POU class 4 homeobox 1*; Prdm15, PR-domain zinc finger protein 15; *prox1*, *prospero homeobox 1*; *rho*, *rhodopsin*; rpe, retinal pigmented epithelium; st., stage; uninj., un-injected; *vsx1*, *visual system homeobox 1*; WT, wild type. Error bars indicate standard errors of the means. **, *p* ≤ 0.01; ***, *p* ≤ 0.001.

Prdm15 MO–injected embryos exhibited a spectrum of anomalies ranging from significantly smaller and/or deformed eyes on the injected side to a complete lack of anterior head structures such as the eyes and parts of the forebrain (Figures 2A, B). A detailed analysis of vibratome sections confirmed a smaller eye size (black arrowheads) and an abnormal eye shape (green arrowheads) and revealed a disordered lamination of the retina (Figure 2A). Notably, some embryos with maldeveloped eyes had no lens. Co-injection of human full-length *PRDM15* (*hPRDM15-WT*) RNA that is not targeted by Prdm15 MO rescued all the above-described Prdm15 MO–induced phenotypes in most embryos. Nevertheless, the successful rescue with *hPRDM15-WT* indicates the specificity of Prdm15 MO–induced phenotypes, implying a conserved function of Prdm15 across species (Figures 2A, B).

To further analyze the retinal lamination and lens structure, we performed whole-mount *in situ* hybridization (WMSH) of well-characterized retina-cell-type marker genes followed by transversal vibratome sectioning (Cizelsky et al., 2013). Prdm15 MO–injected embryos showed a severe retinal lamination phenotype in contrast to CoMO-injected embryos in stage 43 (Figure 2C). Specific marker genes for retina cell layers such as photoreceptor (*rho; rhodopsin*), horizontal (*prox1; prospero homeobox 1*), bipolar (*vsx1; visual system homeobox 1*), amacrine/ganglion (*pax6; paired box 6*), and ganglion (*pou4f1; pou class 4 homeobox 1*) cells displayed a disorganized and displaced localization in the retina upon Prdm15 KD (Figure 2C). Probes specific for the lens marker genes *celf1* (*CUGBP elav-like family member 1*; mature lens fiber cells) and *cryba1* (*crystallin beta A1*; stem cells) labeled smaller areas in Prdm15 morphants, albeit the organization of the cells was unaffected (Figure 2D).

### 2.3 C844Y is pathogenic in anterior neural development in *X. laevis*

**FIGURE 3:**
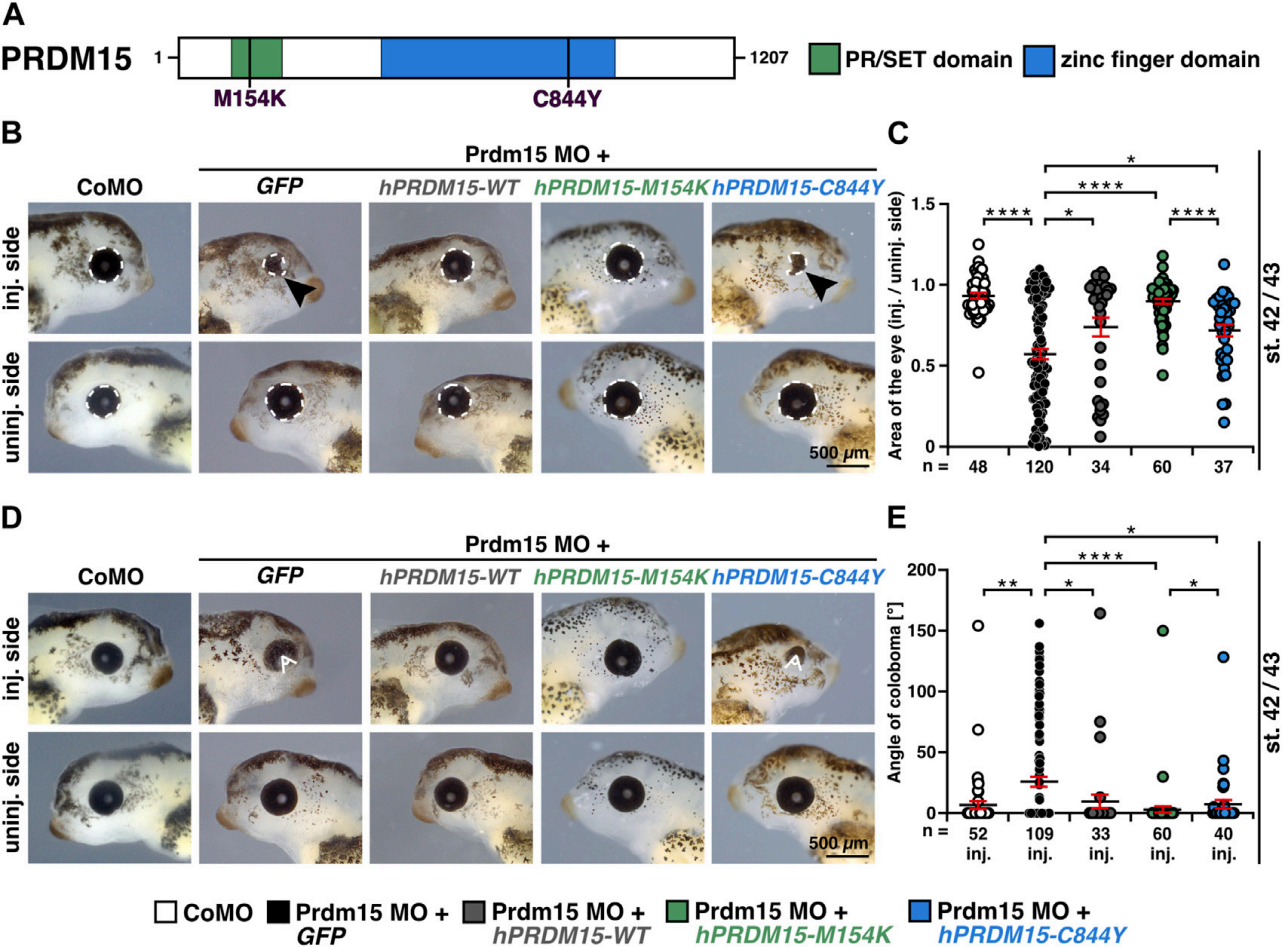
Prdm15 knockdown is rescued by human *PRDM15* RNA with a PR/SET domain mutation but only partially by the *PRDM15* RNA with a zinc finger mutation. **(A)** Schematic overview of the human PRDM15 protein. The PR/SET and zinc finger domains are shown. The positions of the mutated proteins in the PR/SET [p.M154K (c.461T>A); Mann et al., 2021; Mzoughi et al., 2020] and zinc finger domain [p.C844Y (c.2531G>A); Mann et al., 2021; Mzoughi et al., 2020] in GAMOS patients are indicated. **(B)** Co-injection of human full-length *PRDM15-WT* RNA rescues microphthalmia phenotype. While co-injection of human *PRDM15* RNA with a PR/SET variant (*hPRDM15-M154K*) identified in affected individuals also rescues the eye phenotype in most embryos, human *Prdm15* RNA with a variant in the zinc finger domain (*hPRDM15-C844Y*) identified in affected individuals only partially rescues the Prdm15 MO–mediated eye phenotype. Black arrowheads point to smaller and deformed eyes. Representative embryos are shown. **(C)** Statistical evaluation of the eye area as illustrated in **(B)** (white dashed line: measured eye area; injected vs un-injected side). **(D)** Co-injection of human full-length *PRDM15-WT* RNA rescues the coloboma phenotype. Co-injection of *hPRDM15-M154K* RNA rescues the coloboma phenotype in most embryos; *hPRDM15-C844Y* RNA with a variant in the zinc finger domain could partially rescue the Prdm15 MO–mediated coloboma phenotype. White angle shows the measured angle of the colobomas. Representative embryos are shown. **(E)** Statistical evaluation of the coloboma phenotype as illustrated in **(D)** (white angle: measured coloboma angle). Abbreviations: C844Y, cysteine-844-tyrosine; CoMO, control morpholino oligonucleotide; *GFP*, green fluorescent protein; *hPRDM15*, human *PRDM15*; inj., injected; M154K, methionine-154-lysine; MO, morpholino oligonucleotide; n, number of independent experiments; *Prdm15*, PR-domain zinc finger protein 15; st., stage; uninj., un-injected; WT, wild type. Error bars indicate standard errors of the means. *, *p* ≤ 0.05; **, *p* ≤ 0.01; ****, *p* ≤ 0.0001.

This model represents an ideal system for the functional evaluation of the previously reported pathogenic *PRDM15* variant (c.2531G>A, p.C844Y) identified in affected individuals with defective anterior structures (Mzoughi et al., 2020; Mann et al., 2021) (Figure 3A). We, therefore, quantitatively measured the eye size and showed a significantly smaller eye area upon Prdm15 depletion describing a strong microphthalmia phenotype (Figures 3B, C) and a coloboma phenotype (Figures 3D, E), consistent with the ocular defects in patients with HPE or GAMOS (Roessler and Muenke, 1998; Al-Rakan et al., 2018; Lin et al., 2018; Racine and Golden, 2021; Ramakrishnan and Gupta, 2023). These phenotypes could be significantly rescued by *hPRDM15-WT* RNA (Figures 3B–E). While co-injection of human *PRDM15* RNA with a PR/SET domain mutation (*hPRDM15-M154K*) rescued these phenotypes, *PRDM15* RNA with a zinc finger mutation (*hPRDM15-C844Y*) only partially rescued the eye phenotypes (Figures 3B–E). Comparing the two PRDM15 mutation variants, the embryos rescued with *hPRDM15-C844Y* RNA tended to have smaller eyes and more coloboma than the embryos rescued with the *hPRDM15-M154K* variant (Figures 3B–E), supporting the C884Y variant as more pathogenic in the anterior neural development.

### 2.4 Prdm15 is essential for proper eye development in *X. laevis*

**FIGURE 4:**
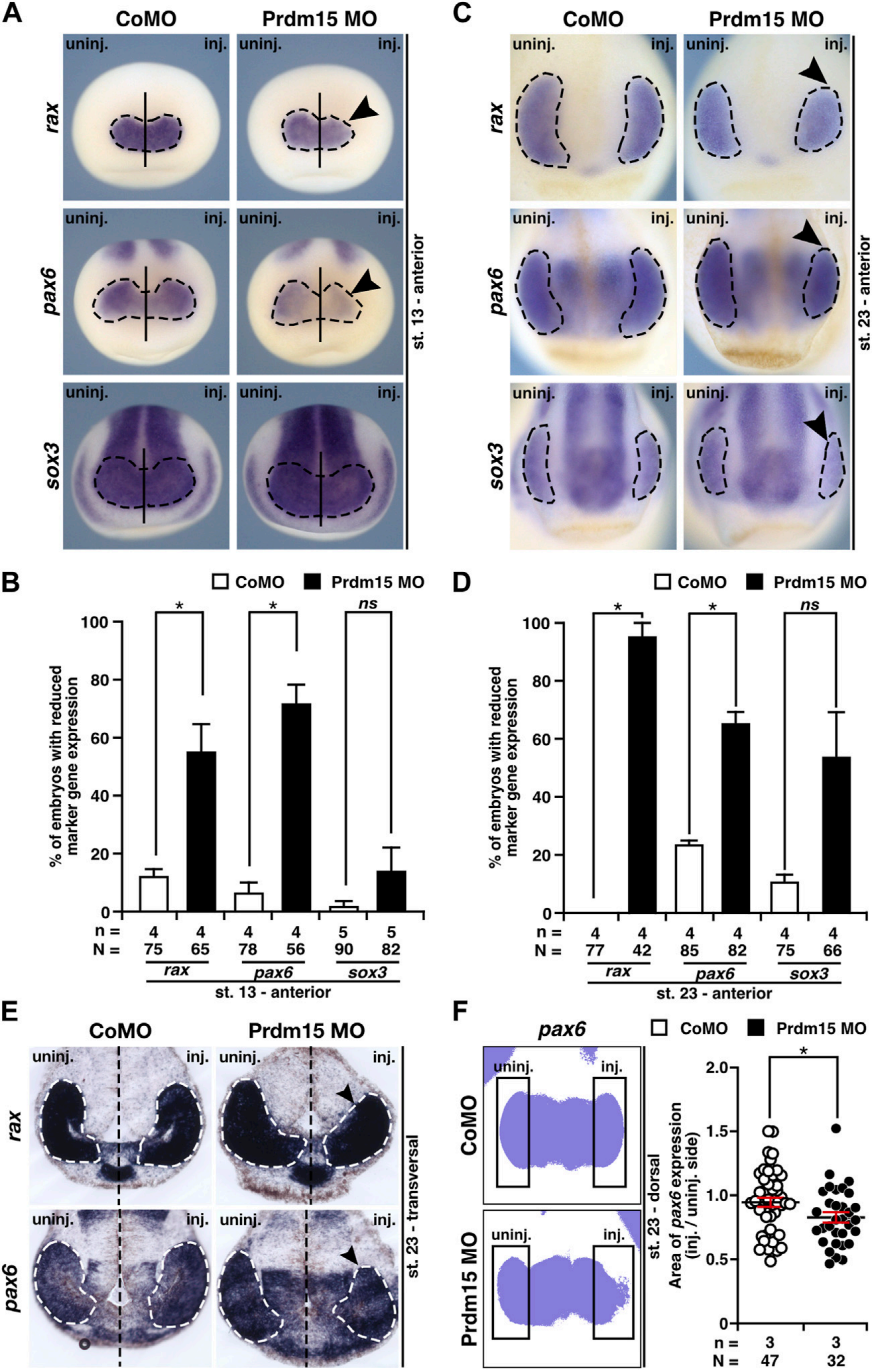
Prdm15 is necessary for proper eye development in *X. laevis*. **(A)** In stage 13, during eye field induction, the anterior expression (black dashed line) of the eye-specific marker genes *rax* and *pax6* is reduced (black arrowheads) in Prdm15 MO–injected embryos visualized by whole-mount *in situ* hybridization (WMISH). By contrast, the pan-neural marker gene *sox3* and CoMO injection did not alter the expression on the injected side. **(B)** Statistical evaluation of embryos with reduced marker gene expression as described in **(A)**. **(C)** Prdm15 knockdown (KD) interferes with eye-specific marker gene expression (black dashed line) of *rax*, *pax6*, and *sox3* in stage 23. Anterior views show a reduced expression in the developing eye (black arrowheads). **(D)** Statistical evaluation of embryos with reduced marker gene expression as described in **(C)**. **(E)** Prdm15 KD influences eye-specific *rax* and *pax6* expressions. Transversal vibratome sections after WMISH with *rax* and *pax6* confirm the eye phenotype showing a smaller and reduced marker gene expression area (black arrowheads). **(F)** Statistical evaluation of embryos with *pax6* expression area as analyzed by ImageJ2 (Rueden et al., 2017) shows a significantly reduced expression area in Prdm15 MO–injected embryos (black arrowhead). Abbreviations: CoMO, control morpholino oligonucleotide; inj., injected; KD, knockdown; MO, morpholino oligonucleotide; n, number of independent experiments; N, number of analyzed embryos in total; *ns*, non-significant; *pax6*, *paired box 6*; Prdm15, PR-domain zinc finger protein 15; *rax*, *retina and anterior neural fold homeobox*; *sox3*, *SRY-box transcription factor 3*; st., stage; uninj., un-injected; WMISH, whole-mount *in situ* hybridization. Error bars indicate standard errors of the means. *ns*, *p* > 0.05; *, *p* ≤ 0.05.

To determine the molecular underpinnings of the lamination defect, we investigated the key steps of eye development in *X. laevis*. During eye field induction at stage 13, the anterior expression of the eye-specific marker genes *rax* (*retina and anterior neural fold homeobox*) and *pax6* was significantly reduced (black arrowheads) in Prdm15 morphants, while the expression of the pan-neural marker *sox3* (*sex-determining region Y-box 3*) remained unaffected (Figures 4A, B). At a later stage (st. 23) during eye cell differentiation, Prdm15 deficiency caused a reduction in the expression of all investigated eye cell–specific marker genes: *rax*, *pax6*, and *sox3* (Figures 4C, D). Consistent with this, both the transversal vibratome sections from *rax* and *pax6* (Figure 4E), as well as the area measurements of one representative experiment of *pax6* (Figure 4F), confirmed the quantified reduced *rax* and *pax6* expression in Prdm15 MO–injected embryos. Furthermore, phospho-histone H3 (pH H3) staining during eye cell differentiation indicated that Prdm15 depletion significantly decreased the number of proliferative cells at the anterior neural tissue on the injected side (Supplementary Figure S2). Taken together, we have shown that Prdm15 is essential for proper eye development and that its depletion already affects early eye field induction in *X. laevis*.

### 2.5 Prdm15 is required for head development in *X. laevis*

**FIGURE 5:**
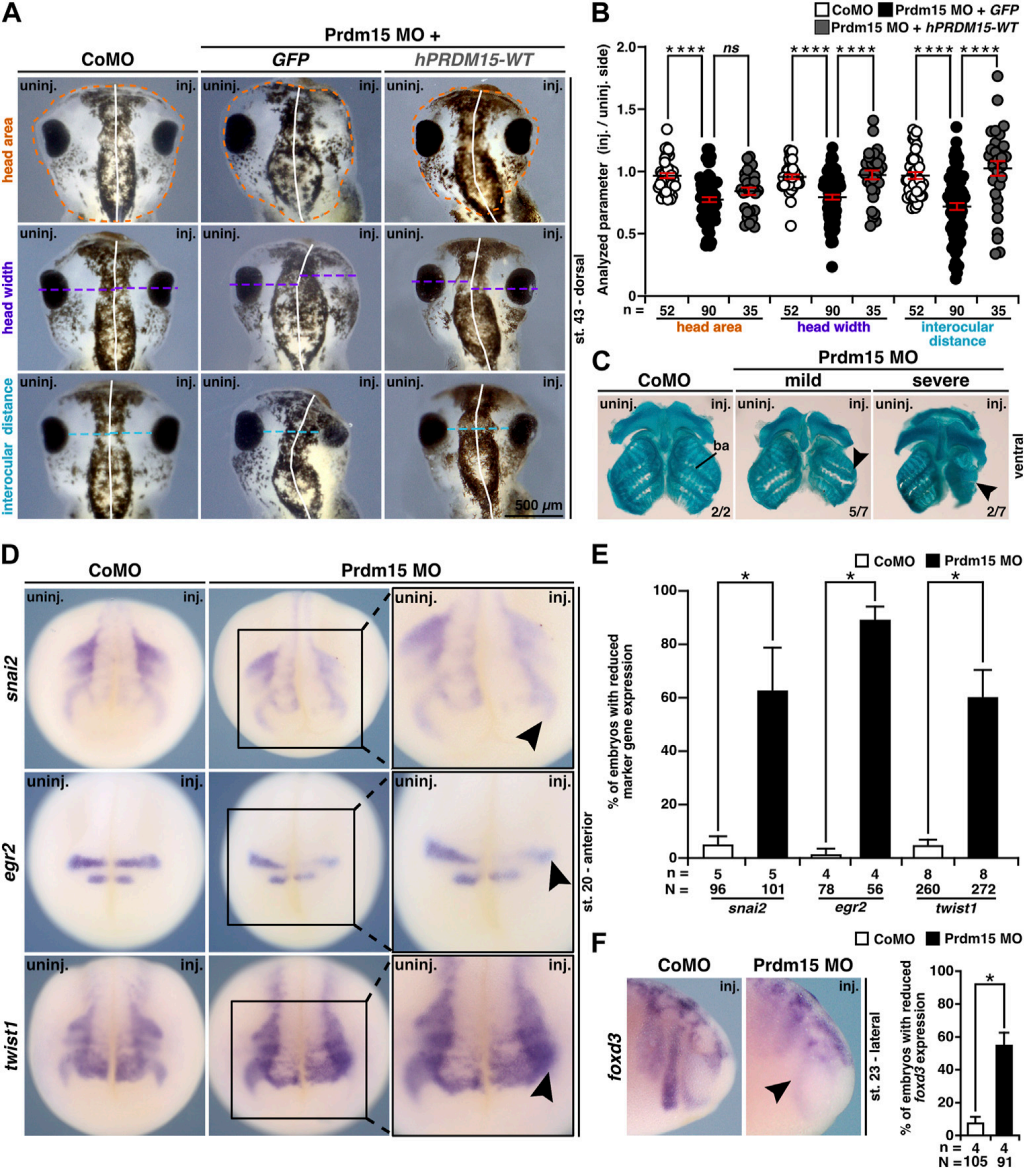
Prdm15 is required for head development in *X. laevis*. **(A)** Unilateral KD of Prdm15 leads to a microcephaly phenotype compared to control MO (CoMO)–injected embryos. Co-injection of human full-length *PRDM15-WT* RNA rescues the head phenotype. The dorsal views of representative stage 43 embryos are shown. Dashed lines indicate the measured area and length. **(B)** Statistical evaluation of smaller and deformed heads as indicated in **(A)** reveals a significantly smaller head area (orange), head width (purple), and interocular distance (light blue), while co-injection of *hPRDM15* RNA rescues almost all Prdm15 MO–induced phenotypes. **(C)** Ventral view of Alcian blue–stained and dissected cranial cartilages from control and Prdm15 morphants (stage 45). Prdm15 KD results in a mild and severe phenotype showing narrowed or deformed cartilage structures (black arrowheads), especially at the branchial arch (ba). **(D)** Anterior view of CoMO-injected and Prdm15 MO–injected embryos (stage 20) after whole-mount *in situ* hybridization (WMISH) with NCC-specific marker genes during NCC migration such as *snai2*, *egr2*, and *twist1* show a reduced marker gene expression on Prdm15 MO–injected side (black arrowheads). **(E)** Statistical evaluation of NCC-specific marker genes' expression as illustrated in **(D)** reveals a significant reduction in marker gene expression upon Prdm15 MO KD. **(F)** Lateral view of CoMO-injected and Prdm15 MO–injected embryos (stage 23) after WMISH with the NCC marker *foxd3* shows a reduced expression on the Prdm15 MO–injected side (black arrowhead). Statistical evaluation of *foxd3* expression reveals a significant reduction in its expression upon Prdm15 MO KD. Abbreviations: CoMO, control morpholino oligonucleotide; *egr2*, *early growth response 2*; *foxd3*, *forkhead box D3*; inj., injected; *hPRDM15*, human *PRDM15*; MO, morpholino oligonucleotide; n, number of independent experiments; N, number of analyzed embryos in total; *ns*, non-significant; Prdm15, PR-domain zinc finger protein 15; *snai2*, *snail family transcriptional repressor 2*; st., stage; *twist1*, *twist family bHLH transcription factor 1*; uninj., un-injected; WMISH, whole-mount *in situ* hybridization; WT, wild type. Error bars indicate standard errors of the means. *ns*, *p* > 0.05; *, *p* ≤ 0.05; ****, *p* ≤ 0.0001.

In addition to the abnormal eye development, Prdm15 MO–injected *X. laevis* embryos exhibited head malformations (Figures 5A, B). To analyze the head phenotype upon Prdm15 KD in more detail, we measured the head area, head width, and interocular distance of *X. laevis* embryos (Figures 5A, B). Prdm15 MO–mediated KD showed a significant reduction in the analyzed parameters that was mainly significantly rescued by co-injection of the human full-length *PRDM15-WT* RNA (Figures 5A, B).

These results prompted us to investigate the cartilage of Prdm15 morphants. Alcian blue staining at the late tadpole stages indicated a spectrum of malformations of the cranial cartilages, in particular a reduction of the branchial arch cartilage in Prdm15 MO–injected embryos (Figure 5C).

Since *prdm15* expression was found in NCCs (Figures 1E, F, I ,J), and NCCs contribute to the formation of the cranial cartilage, we investigated embryos in stage 20 when NCCs migrate. All the three analyzed genes, namely, *snai2* (*snail family transcriptional repressor 2*), *egr2* (*early growth response 2*), and *twist1* (*twist family bHLH transcription factor 1*), were severely reduced in expression upon Prdm15 MO KD (black arrowheads) (Figures 5D, E). In a later stage (st. 23), we showed a significant reduction in NCC-specific marker gene expression of *foxd3* (*forkhead box D3*) (Figure 5F). To summarize, Prdm15 depletion affects NCC development and might contribute to craniofacial defects in *Xenopus laevis*.

### 2.6 Prdm15 is important for cranial nerve and brain development in *X. laevis*

**FIGURE 6:**
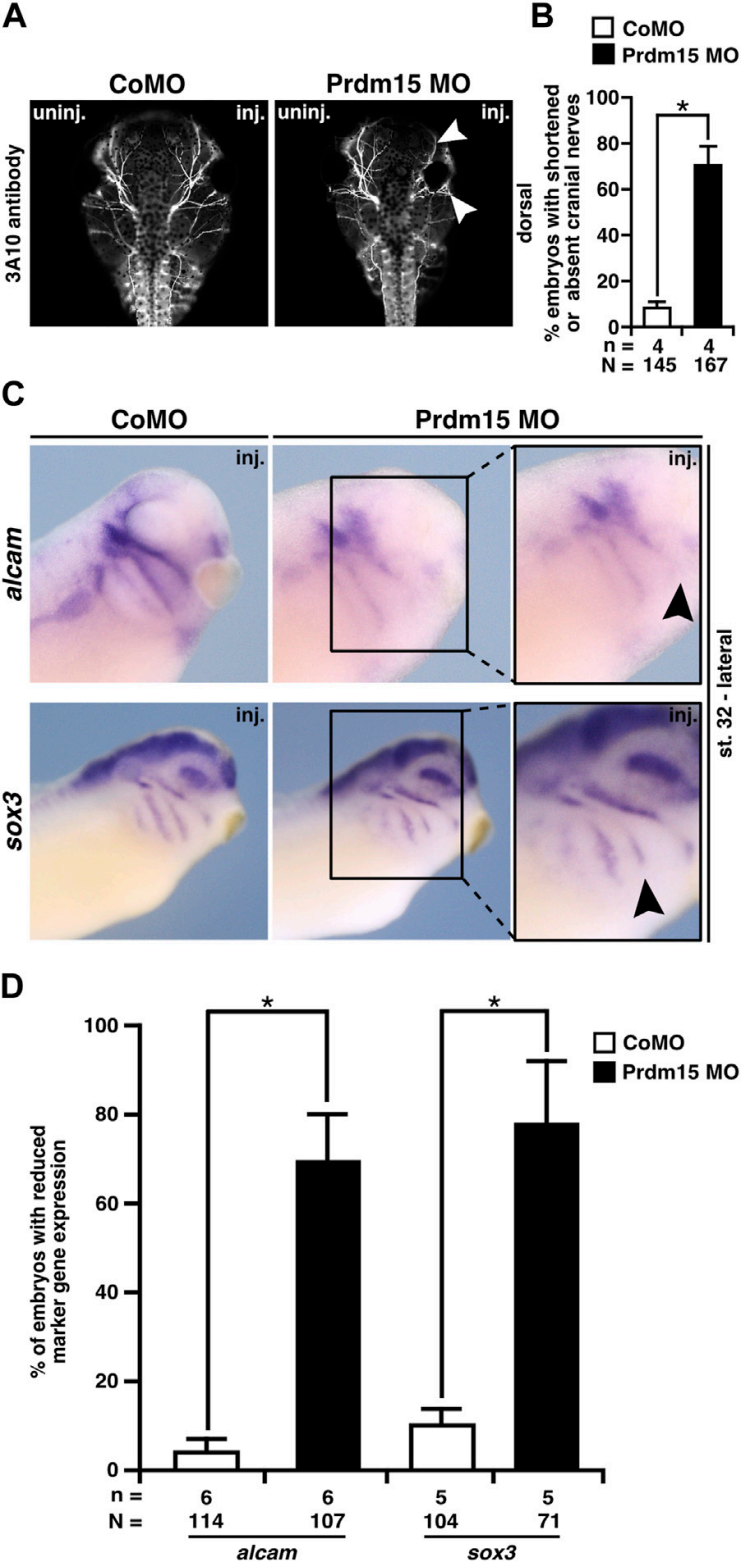
Prdm15 MO injection hinders proper development of the cranial nerves and placodes. **(A)** Dorsal views of control MO-injected and Prdm15 MO–injected embryos show a shortened or decreased branching of cranial nerves (white arrowheads) upon Prdm15 KD visualized by 3A10 antibody staining. **(B)** Statistical evaluation of embryos with shortened or absent branching of cranial nerves as illustrated in **(A)**. **(C)** Lateral view of stage 32 embryos reveals a reduction (black arrowheads) of the respective expression upon Prdm15 MO KD in both marker genes of the lateral placodes (*alcam* and *sox3*) using whole-mount *in situ* hybridization. **(D)** Statistical evaluation of *alcam* and *sox3* expression revealed a significant reduction in its expression upon Prdm15 KD as illustated in **(C)**. Abbreviations: *alcam*, *activated leukocyte cell adhesion molecule*; inj., injected; MO, morpholino oligonucleotide; n, number of independent experiments; N, number of analyzed embryos in total; Prdm15, PR-domain zinc finger protein 15; *sox3*, *SRY-box transcription factor 3*; uninj., un-injected. Error bars indicate standard errors of the means. *, *p* ≤ 0.05.

Since Prdm15 KD contributes to the malformation of the cranial cartilage, we investigated the cranial nerves as another derivate of NCCs. 3A10 antibody staining revealed a clear shortening and/or disorganization of the cranial nerve branches upon Prdm15 KD (Figures 6A, B).

To analyze the molecular basis of the described phenotypes, we investigated the expression of genes that are important for the development of the placodes using the WMISH approach at stage 32. These analyses showed a significant reduction in gene expression of *alcam* that is expressed in the neurogenic placodes (antero/dorsal lateral line placode and profundal–trigeminal placodal area) and *sox3* that is expressed in the lateral line placodes (Figures 6C, D).

Previous studies have shown that Prdm15 loss of function also leads to brain malformations (Mzoughi et al., 2020). To confirm the Prdm15 deficiency in *X. laevis* brain development, we isolated the brains of Prdm15 morphants (stage 42/43) showing a significantly smaller brain area on the injected side (Supplementary Figure S3A). Afterward, we performed a WMISH with several brain-specific genes, such as *rgma* (*repulsive guidance molecule a*; forebrain and midbrain), *otx2* (*orthodenticle homeobox 2*; forebrain and midbrain), *pax6* (forebrain and posterior neural tube), and *egr2* (hindbrain) in Prdm15 MO–manipulated embryos at stages 13 and 23. These analyses revealed a reduced marker gene expression mainly in the midbrain and hindbrain (black arrowheads) (Supplementary Figures S3C, D) and mildly in the forebrain (white arrowheads) (Supplementary Figures S3C–D). To clarify whether there is a reduced expression in the forebrain, we used a further marker gene *emx1* (*empty spiracles homeobox 1*; forebrain). Thus, *emx1* expression is not significantly reduced on the Prdm15 MO–injected side (Supplementary Figure S3E). In addition, *tubb2b* that is one of the first genes expressed in primary neurons of the neural plate showed a significant reduced expression in respective dorsal neural tissue (Supplementary Figure S4).

Taken together, these data indicate that in addition to anterior/posterior patterning defects (Mzoughi et al., 2020), Prdm15 depletion affects placode and brain cell differentiation and could contribute to head and brain defects in *X. laevis*.

### 2.7 Prdm15 acts upstream of Wnt4

We previously showed that Prdm15 is an important factor for early pronephros (Mann et al., 2021), eye, head, and brain development. Additionally, we noted a defective WNT/PCP signaling in PRDM15 mutant mice, which exhibited patterning defects resulting in brain malformations (Mzoughi et al., 2020). The PRDM15 transcriptional target gene Wnt4 is of particular interest given its crucial role in kidney and eye development (Maurus et al., 2005; Cizelsky et al., 2014).

**FIGURE 7:**
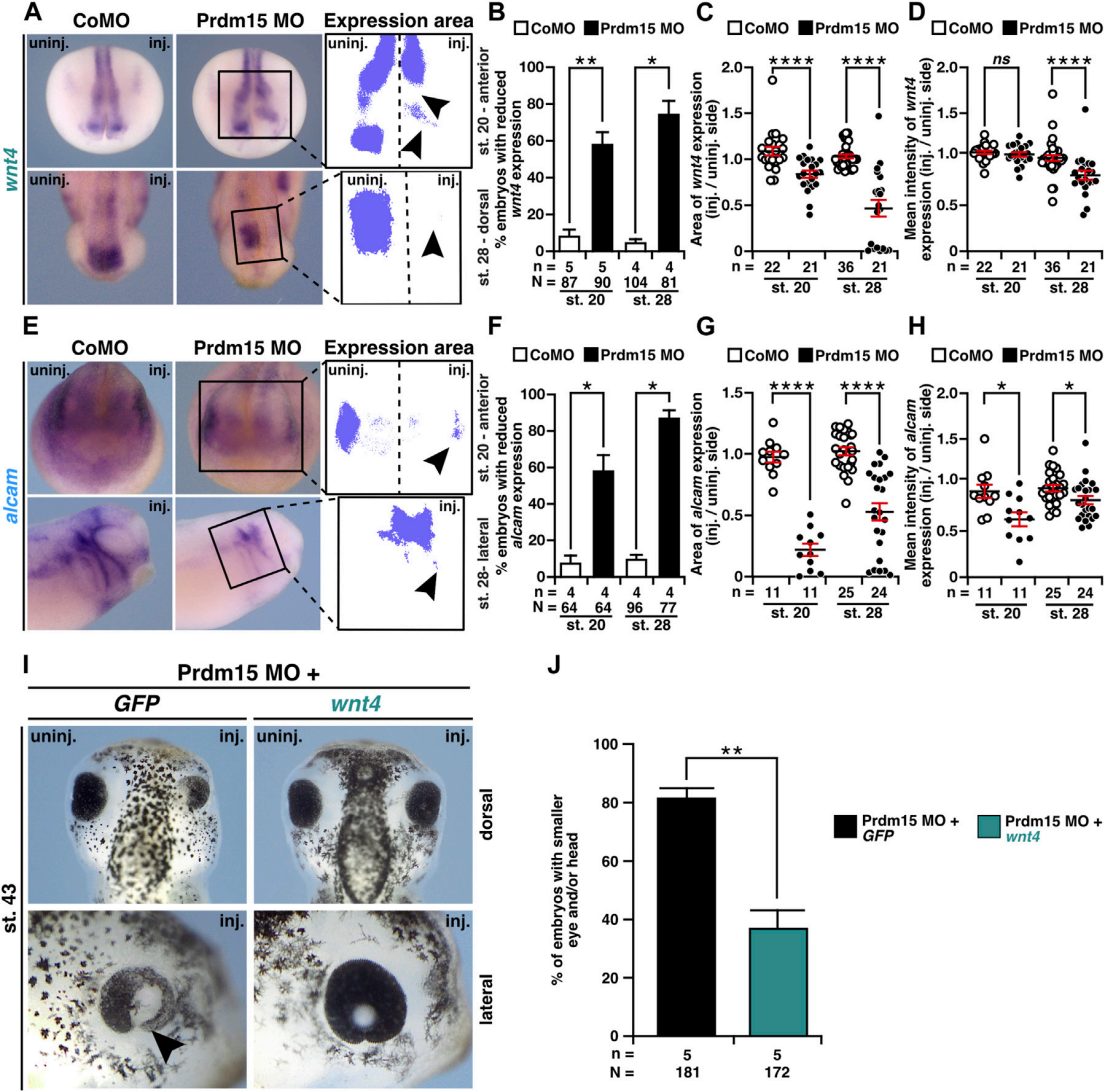
Prdm15 acts upstream of Wnt4. **(A)** Anterior view at stage 20 and dorsal view at stage 28 of Prdm15 MO–injected embryos show a reduced expression of *wnt4* (black arrowheads) in comparison to the un-injected side, as analyzed by the expression area and intensity of *wnt4* after whole-mount *in situ* hybridization (WMISH). **(B)** Statistical analysis of embryos with reduced *wnt4* expression as indicated in **(A)** analyzed with a light microscope. **(C)** Statistical analysis of the expression area of *wnt4* as described in **(A)** using a computer-based approach. **(D)** Statistical analysis of the mean intensity of the expression area of *wnt4* as described in **(A)** using a computer-based approach. The statistical analysis of *wnt4* expression reveals a significantly reduced *wnt4* expression except for the analysis of *wnt4* in stage 20 upon Prdm15 knockdown. **(E)** Anterior view of stage 20 and lateral view of stage 28 embryos. Prdm15 MO–injected embryos show a reduction in *alcam* expression on the injected side (black arrowheads), as analyzed by expression area **(G)** and intensity **(H)** using WMISH. **(F)** Statistical analysis of embryos with reduced *alcam* expression analyzed with a light microscope. **(G)** Statistical analysis of the expression area using a computer-based approach. **(H)** Statistical analysis of the mean intensity of the expression area of *alcam* using a computer-based approach reveals a significantly reduced *alcam* expression upon Prdm15 knockdown. **(I)** Dorsal and lateral views of stage 43 embryos show rescue of the Prdm15 MO–induced head and eye phenotype by *wnt4* RNA. Black arrowhead points to the reduced and deformed eye size upon Prdm15 MO injection. **(J)** Statistical evaluation of the embryos with smaller eye and/or head as illustrated in **(I)**. Abbreviations: *alcam*, *activated leukocyte cell adhesion molecule*; CoMO, control morpholino oligonucleotide; *GFP*, green fluorescent protein; inj., injected; MO, morpholino oligonucleotide; n, number of independent experiments; N, number of analyzed embryos in total; *ns*, non-significant; Prdm15, PR-domain zinc finger protein 15; st., stage; uninj., un-injected; WMISH, whole-mount *in situ* hybridization; *wnt4*, *wnt family member 4*. Error bars indicate standard errors of the means. *ns*, *p* > 0.05; *, *p* ≤ 0.05; **, *p* ≤ 0.01; ****, *p* ≤ 0.0001.

To investigate whether Wnt4 is relevant to the congenital malformations caused by PRDM15 depletion, we first monitored *wnt4* expression upon Prdm15 KD. WMISH with a *wnt4* probe indicated a significant reduction in *wnt4* expression area and intensity in Prdm15 morphants (Figures 7A–D). Moreover, the expression of the direct Wnt4 downstream target gene *alcam* was also significantly reduced upon Prdm15 MO injection (Figures 7E–H). More importantly, injection of *wnt4* RNA partially rescued the eye and head phenotypes induced by PRDM15 MO KD (Figures 7I, J), supporting the hypothesis that Prdm15 is upstream of *wnt4*.

**FIGURE 8:**
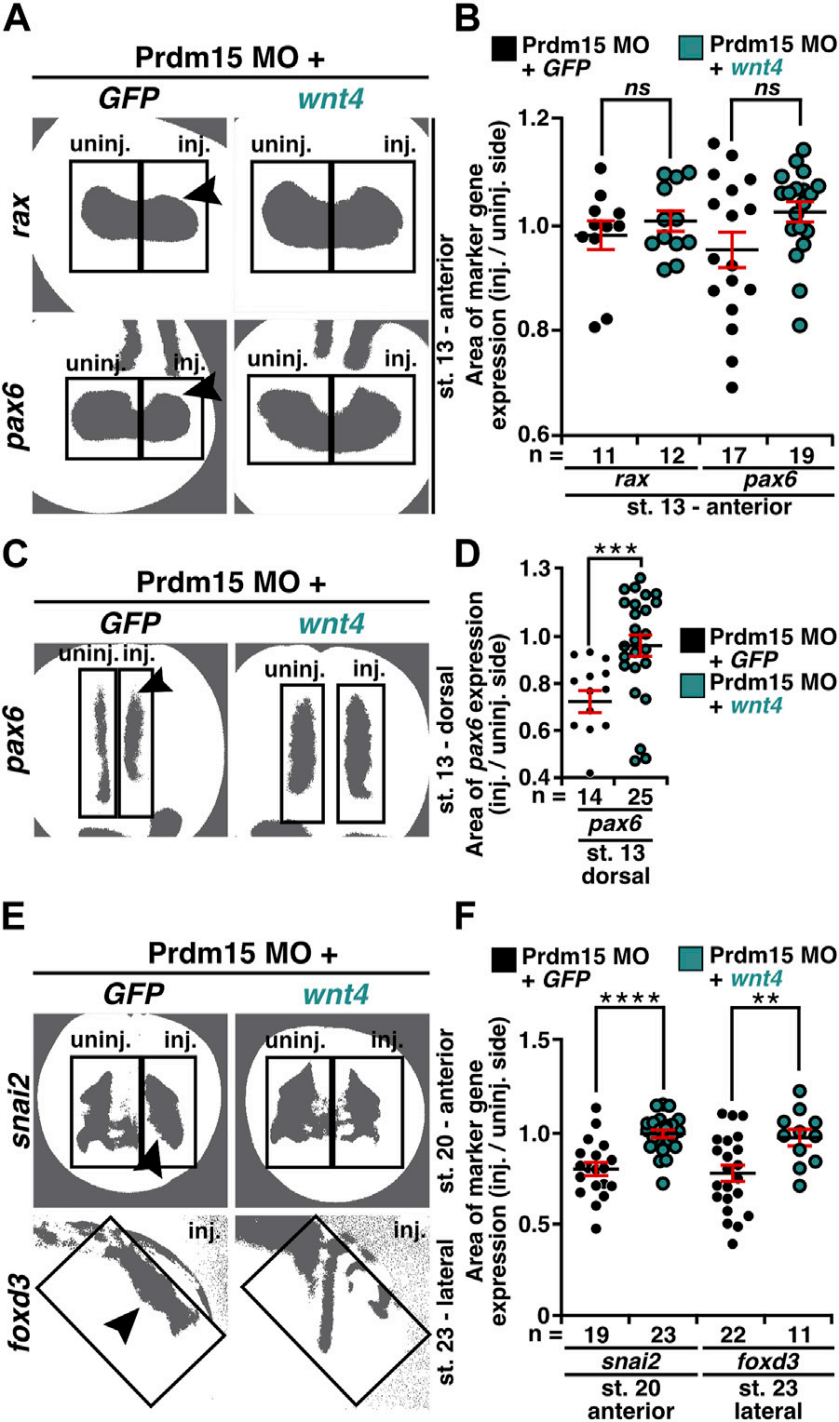
Co-injection of *wnt4* RNA rescues the Prdm15 MO–induced reduced gene expression. **(A)** Anterior view of stage 13 embryos injected with Prdm15 MO in combination with *wnt4* RNA refers to the measured *rax* and *pax6* expression area. Black arrowheads point to the reduced gene expression. *Wnt4* RNA could partially rescue the Prdm15 MO–induced reduced marker gene expression. **(B)** Statistical evaluation of the marker gene expression area of *rax* and *pax6* as described in **(A)**. **(C)** Dorsal view of stage 13 embryos injected with Prdm15 MO in combination with *wnt4* RNA illustrates the measured *pax6* expression area. Black arrowhead points to the reduced gene expression. Co-injection of *wnt4* RNA restores the Prdm15 MO–induced *pax6* expression. **(D)** Statistical evaluation of the marker gene expression area as described in **(C)** shows rescue of the dorsal *pax6* expression by *wnt4* RNA. **(E)** Anterior and lateral views of stage 20 and stage 23 embryos, respectively, injected with Prdm15 MO in combination with *wnt4* RNA illustrate the measured *snai2* and *foxd3* expression area. Black arrowheads point to the reduced gene expression. Co-injection of *wnt4* RNA rescues the Prdm15 MO–induced reduced marker gene expression. **(F)** Statistical evaluation of *snai2* and *foxd3* expression area as described in **(E)** shows a significant rescue of the expression area of *snai2* and *foxd3*. Abbreviations: *foxd3*, *forkhead box D3*; *GFP*, green fluorescent protein; inj., injected; MO, morpholino oligonucleotide; n, number of independent experiments; *ns*, non-significant; *pax6*, *paired box 6*; Prdm15, PR-domain zinc finger protein 15; *rax*, *retina and anterior neural fold homeobox*; *snai2*, *snail family transcriptional repressor 2*; st., stage; uninj., un-injected; *wnt4*, *wnt family member 4*. Error bars indicate standard errors of the means. *ns*, *p* > 0.05; **, *p* ≤ 0.01; ***, *p* ≤ 0.001; ****, *p* ≤ 0.0001.

As we observed that Prdm15 KD reduced the expression of early marker genes (Figures 4–6 and Supplementary Figures S3, S4), we wondered whether these phenotypes could be rescued by *wnt4* RNA. Thus, we co-injected Prdm15 MO together with *wnt4* RNA and analyzed the expressions of *rax*, *pax6*, *snai2*, and *foxd3* (Supplementary Figures S5A–F and Figures 8A–F). WMISH with the eye-specific genes *rax* and *pax6* indicated a partial rescue of their anterior expression at stage 13 upon co-injection of Prdm15 MO together with *wnt4* RNA (Supplementary Figures S5A, B). The detailed expression area analysis of one representative experiment confirmed this partial rescue of *rax* and *pax6* expressions at stage 13 (Figures 8A, B). In addition, *pax6* expression analysis in the dorsal tissue showed a partial rescue by *wnt4* RNA at stage 13 (Supplementary Figures S5C, D). A detailed analysis by quantitative measurement of the expression area of one representative experiment revealed a significant rescue of *pax6* expression upon injection of *wnt4* RNA at stage 13 (Figures 8C, D). At later stages (20 and 23), the microscopic analysis of *snai2* and *foxd3* expressions showed a partial rescue by *wnt4* RNA (Supplementary Figures S5E, F), while the quantitative analysis of *snai2* and *foxd3* expression areas by measurement of one representative experiment even revealed a significant rescue of their expressions (Figures 8E, F). To sum up, these data suggest Prdm15 to be upstream of Wnt4 during the early anterior neural development in *X. laevis*.

### 2.8 Prdm15 influences both canonical and non-canonical Wnt signaling

**FIGURE 9:**
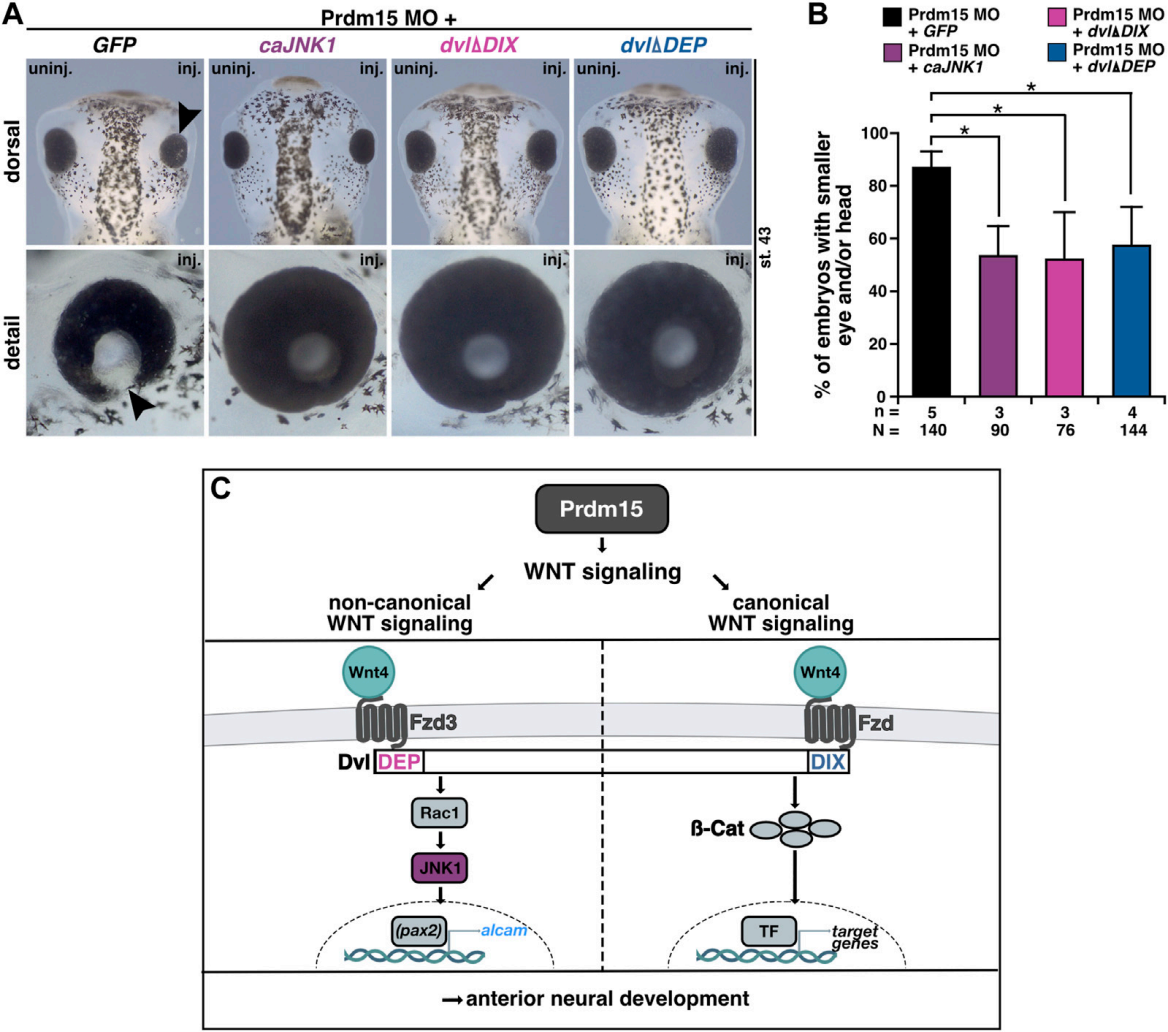
Prdm15 influences canonical and non-canonical Wnt signaling upstream of Wnt4. **(A)** Dorsal and detailed view of stage 43 embryos injected with Prdm15 MO in combination with *GFP* RNA or the constitutive active *JNK1* (*caJNK1*) RNA or the two different mutated disheveled versions (*dvlΔDIX*, *dvlΔDEP*). The co-injection of Prdm15 MO with *caJNK1* and the *dvlΔDIX*-mutated version as well as the *dvlΔDEP*-mutated version shows rescue of the severe Prdm15 MO–induced phenotype in contrast to the negative control *GFP* (black arrowhead). **(B)** Statistical evaluation of embryos with smaller eye or head as described in **(A)** shows a rescue with *caJNK1*, *dvlΔDIX*, and *dvlΔDEP* RNA. **(C)** Schematic overview of Prdm15 and WNT signaling. Prdm15 acts upstream of Wnt4 and influences the expression of *wnt4* and *alcam* through the non-canonical WNT/PCP signaling pathway possibly via the transcription factor *pax2*. Furthermore, the canonical Wnt signaling pathway is also affected downstream of Prdm15. Abbreviations: *alcam*, *activated leukocyte cell adhesion molecule*; *ß-cat,* β*-catenin*; *caJNK1*, *constitutive active JNK 1*; *DEP*, disheveled *Egl-10* and *pleckstrin*; *DIX*, *disheveled Axin*; *dvl*, *disheveled*; Fzd, frizzled; Fzd3, frizzled3; *GFP*, green fluorescent protein; inj., injected; JNK1, c-Jun N-terminal kinase 1; MO, morpholino oligonucleotide; n, number of independent experiments; N, number of analyzed embryos in total; *pax2*, *paired box 2*; Prdm15, PR-domain zinc finger protein 15; Rac1, Rac1 family small GTPase 1; st., stage; TF, transcription factor; uninj., un-injected; WNT, wingless-type MMTV integration site family member; *wnt4*, wnt family member 4. Error bars indicate standard errors of the means. *, *p* ≤ 0.05.

PRDM15 is known to regulate both canonical WNT/β-catenin and non-canonical WNT/PCP signaling pathways (Mzoughi et al., 2017; Mzoughi et al., 2020). We thus sought to investigate whether Prdm15-mediated regulation of the canonical or non-canonical Wnt signaling pathway is implicated in the observed phenotypes. In the end, we injected Prdm15 MO in combination with either the constitutive active version of *JNK1* (*caJNK1*) or one of the two well-characterized disheveled (dvl) deletion constructs (*dvlΔDIX*, *dvlΔDEP*) (Kishida et al., 1999; Li et al., 1999; Miller et al., 1999; Cizelsky et al., 2014). Both *caJNK1* and *dvlΔDIX* activate the non-canonical WNT/PCP pathway, whereas *dvlΔDEP* activates the canonical Wnt signaling pathway since the DIX domain is still available to recruit Axin to the receptor complex (Figure 9). Interestingly, *caJNK1* and the two different mutated dvl versions significantly rescued the Prdm15 MO–induced phenotype, suggesting a prominent role of both pathways (Figure 9).

## 3 Discussion

### 3.1 Prdm15 conservation and expression in disease-relevant tissues

In our study, we performed the first synteny analysis with *Homo sapiens*, *Mus musculus*, *Xenopus laevis*, *Xenopus tropicalis*, and *Danio rerio* (*D. rerio*), showing a high similarity in neighboring gene loci. *In silico* analysis of *prdm15* identified a strong evolutionary conservation. We expanded our previous study (Mann et al., 2021) and included the species *X. laevis*, revealing a high homology of Prdm15 protein alignment across species and even a stronger evolutionary conservation within the domains.

To date, there have been two studies on the expression pattern of *prdm15* during early embryonic development in *D. rerio* and *X. laevis* (Sun et al., 2008; Eguchi et al., 2015). In embryos of *D. rerio*, *prdm15* expression was observed in cranial ganglia neurons and in muscle pioneer cells during three time points in early embryonic development (Sun et al., 2008), while in *X. laevis*, *prdm15* transcripts were detected in the developing eye, branchia, and brain during organogenesis (Eguchi et al., 2015). At the transcriptional level, *prdm15* has shown activity throughout the early embryonic development of *X. laevis* (Eguchi et al., 2015; Session et al., 2016). Consistent with the previous expression analysis, our study provides the first highly detailed spatiotemporal expression pattern analysis in a vertebrate organism during early development. We showed an enrichment of transcripts in the structures of the developing brain, eye, head, and pronephros, tissues and organs that are also affected in GAMOS patients. Interestingly, the expression of other GAMOS-related genes such as *osgep*, *tp53rk*, and *tprkb* has a very similar expression pattern during *X. laevis* development (Treimer et al., 2021). Additionally, there is also an expression of HPE-associated genes (*six3*, *tgif1*, *gli2*, *lrp2*, *smad2*, and *smad4*) in the developing eye and forebrain (Ghanbari et al., 2001; Perron et al., 2003; Zaghloul and Moody, 2007; Kerr et al., 2008; Zhang et al., 2017; Kowalczyk et al., 2021), pointing to a converging expression area in the developing embryo. This expression analysis indicates a potential function of Prdm15 in the development of the central nervous system of *X. laevis* and in GAMOS.

### 3.2 Prdm15 depletion recapitulates anterior neural anomalies in syndromic HPE

Our phenotypic characterization revealed a defect in A/P patterning occurring in a portion of embryos lacking anterior structures such as eyes and parts of the forebrain and head, consistent with previous findings in mouse embryos (Mzoughi et al., 2020).

In this study, we focused on the severe eye phenotypes from anophthalmia, microphthalmia, to coloboma in combination with an impaired retinal lamination in *X. laevis*, recapitulating the wide spectrum of ocular anomalies from HPE and GAMOS patients (Roessler and Muenke, 1998; Lin et al., 2018; Racine and Golden, 2021; Ramakrishnan and Gupta, 2023). Additionally, MO-based Prdm15 KD also leads to a smaller head size and defects in cranial cartilage and nerve development, recapitulating the anterior neural anomalies in syndromic HPE. Co-injection of human *PRDM15-WT* RNA rescued these severe eye and head phenotypes.

Thus, our model system is ideal for the functional evaluation of previously reported pathogenic *PRDM15* variants identified in affected individuals with defective anterior structures (Mzoughi et al., 2020; Mann et al., 2021). While co-injection of human *PRDM15* RNA with a PR/SET domain mutation (*hPRDM15-M154K*) rescued the eye phenotypes in most of the embryos, *PRDM15* RNA with a zinc finger mutation (*hPRDM15-C844Y*) only partially rescued these phenotypes. The PR/SET domain variant showed fewer embryos with severe phenotypes than the zinc finger mutation, supporting the C884Y variant as more pathogenic in anterior neural development. GAMOS is a syndromic disease and one severe clinical aspect affects the kidneys. The associated disorder is the steroid-resistant nephrotic syndrome (SRNS). Previously, we demonstrated that *prdm15* is expressed during renal embryonic development, and its deficiency leads to developmental failure in *X. laevis* (Mann et al., 2021). In the embryonic kidney, none of the *PRDM15* patients' mutations (M154K, E190K, and C844Y) rescued the renal phenotype by co-injecting RNA, showing that all mutations are pathogenic and relevant in developing SRNS (Mann et al., 2021). We also aimed to investigate the potential causes for these different phenotypes in different organs (brain, head, and kidneys) to find a converging mechanism. We can only speculate that Prdm15 could be a possible connector in regulating different key signaling pathways that are important for the development of the brain, head, and kidneys. Of note, we could also report a severe head and eye phenotype in another GAMOS-associated gene, *tp53rk* (Treimer et al., 2022), but if there is a hierarchical order or a common molecular mechanism in GAMOS remains to be investigated. Consequently, it is essential to investigate the exact function, converging molecular mechanisms, and signaling networks of Prdm15- and GAMOS-associated genes in the developing central nervous system.

### 3.3 Prdm15 influences crucial steps of anterior neural development

Our findings showed that crucial developmental steps of eye, head, and brain development were disrupted upon Prdm15 depletion. We investigated several important developmental marker genes during *X. laevis* eye, brain, and NCC development.

Since NCCs contribute to the formation of diverse cell lineages and structures, such as the peripheral nervous system, craniofacial skeleton, smooth muscle, skin pigmentation, and multiple ocular and periocular structures (Le Douarin and Dupin, 2018), abnormalities in NCC development cause craniofacial defects and ocular anomalies (Williams and Bohnsack, 2015; Siismets and Hatch, 2020). Our results indicate that Prdm15 depletion has a strong influence on NCC development by reducing important NCC-specific marker genes such as *snai2*, *egr2*, *twist1*, and *foxd3* upon Prdm15 KD. These early defects lead to disturbed cranial cartilages and nerves observed in Prdm15-deficient embryos. It has been previously elucidated that some key transcriptional regulators, such as *foxd3* or *snai2*, play an important role when neural differentiation occurs (Méndez-Maldonado et al., 2020). As Prdm15 was found to act as a transcription factor, it could be involved in the first steps of NCC development.

NCCs were shown to play also a critical role in the maintenance of gene expression that is important for forebrain development. In our study, the expression of important brain-specific marker genes *pax6*, *otx2*, and *egr2* was reduced upon Prdm15 depletion.

Additionally, we investigated eye-related marker genes like *pax6*, showing that a reduction of their expressions during eye field induction and at eye cell differentiation upon Prdm15 KD might lead to severe eye phenotypes. Most likely, an early disruption of eye development contributes to a disturbed eye cup invagination resulting in maldevelopment of the eye. *Pax6* depletion in mice leads to absent eyes (Georgala et al., 2011), which we also observed in a few Prdm15 MO–manipulated embryos. Consistent with our findings upon Prdm15 KD, *PAX6* gene mutations in humans are associated with eye defects such as aniridia and corneal opacification or cataract (Cvekl and Callaerts, 2017). In our study, Prdm15 KD leads to a reduced *rax* expression. Previously, it was shown that deletion of *rax* affects proper retinal lamination since *rax* might be necessary for retinal progenitor proliferation and cell fate specification in mouse embryos (Rodgers et al., 2018). In addition, *rax*-dependent genes such as *neil3* play a role in proper retinal lamination in *X. laevis* (Pan et al., 2018). Not only *PAX6* but also *OTX2* and *EGR2* have been associated with eye and brain defects in humans (Gonzalez-Rodriguez et al., 2010; Nakayama et al., 2015; Sevilla et al., 2015; Deml et al., 2016). Minor proliferation at the retina could also be a reason for smaller eye size, and indeed, we found that Prdm15 depletion results in less proliferative cells in the anterior neural tissue. In our study, lens marker genes were only affected in size and not in their composition upon Prdm15 depletion, suggesting that in this case, a disturbed development of the lens placodes does not influence the disorganized lens structure. The pan-neural maker gene *sox3*, however, was not affected at stage 13, indicating that neural induction is not disturbed in general.

Taken together, these data indicate that Prdm15 KD leads to a disturbed expression of important key developmental genes, resulting in a severe eye, brain, and head phenotype. It can be assumed that Prdm15 acts on various early events during embryogenesis and during the development of anterior neural structures.

### 3.4 Prdm15 acts upstream of canonical and non-canonical Wnt

Previously, we have found that a defect in Wnt4 and its direct target gene *alcam* can lead to eye and kidney maldevelopment (Maurus et al., 2005; Cizelsky et al., 2014; Seigfried et al., 2017). Former studies have also shown that non-canonical Wnt signaling is especially required for early vertebrate eye development (Maurus et al., 2005; Gessert et al., 2007; Bugner et al., 2011). Moreover, JNK1 has already been linked to defects in the closure of the optic fissure (Weston et al., 2003). Our study revealed that Prdm15 KD leads to a reduced expression of *wnt4* and *alcam* RNA in anterior neural structures. More importantly, the Prdm15 MO KD phenotype could be significantly rescued by co-injection of *wnt4* RNA, indicating that Prdm15 acts upstream of Wnt4. Interestingly, also the Prdm15 MO–mediated reduced expression of some key factors such as *rax*, *pax6*, *snai2*, and *foxd3* could be rescued by *wnt4* RNA, indicating that Wnt4 has already an important role during the first developmental steps in *X. laevis*. However, the partial nature of these rescues may imply that concomitant restoration of defective NOTCH signaling is required to achieve a more complete rescue due to the fact that Prdm15 loss of function links NOTCH and WNT/PCP to patterning defects in HPE (Dupé et al., 2011; Mzoughi et al., 2020). Since Prdm15 depletion could influence the expression of *Rspo1* in mESCs and Wnt4 could affect canonical WNT signaling by switching between two modes of β-catenin function—transcriptional activation and cell–cell adhesion—Wnt4 could be involved in both canonical and non-canonical signaling in a context-dependent manner (Bernard et al., 2008). Therefore, we additionally investigated the role of Prdm15 in the canonical Wnt signaling pathway. By using *caJNK1* or *dvlΔDIX* and *dvlΔDEP* RNA, we uncovered that Prdm15-mediated KD influences both canonical and non-canonical WNT/PCP signaling during *X. laevis* early anterior neural development.

In conclusion, our data provide an important overview of *prdm15* expression during early embryogenesis. We showed that *prdm15* transcripts are specifically expressed in the developing brain, eye, and NCCs. In this study, using *X. laevis* embryos, we validated brain malformations observed in humans and mice and provided deeper mechanistic insights into ocular abnormalities. We demonstrate that both eye and head defects caused by Prdm15 depletion can be rescued by co-injection of human *PRDM15-WT* RNA. While a PR/SET domain (c.461T>A; p.M154K) mutant rescued the severe eye phenotypes in most embryos, the zinc finger (c.2531G>A; p.C844Y) mutant could only partially rescue these phenotypes, confirming the pathogenic potential of the C844Y variant. At the molecular level, we uncovered that Prdm15 acts upstream of both canonical and non-canonical Wnt signaling during *X. laevis* embryogenesis, presumably through the regulation of *wnt4* transcription.

Prdm15 represents a central point to coordinate early signaling processes such as canonical and non-canonical WNT/PCP signaling to facilitate key events during embryonic anterior neural development. Furthermore, Prdm15 acts as a master regulator of several crucial pathways in early development, i.e., WNT, SHH, NOTCH, NODAL, and MAPK, in a context-dependent manner, but the exact mechanistic details remain to be investigated.

## 4 Materials and methods

### 4.1 Synteny analysis and protein alignment of Prdm15

Synteny analysis of *prdm15* was performed by comparing the gene location between the species: human, mouse, frog, and fish using the NCBI GenBank (https://www.ncbi.nlm.nih.gov/genbank/). To determine the PR/SET and zinc finger domains in the different species, the ScanProsite tool was used (https://prosite.expasy.org/scanprosite/) (de Castro et al., 2006). Afterward, multiple sequence alignments and calculations of the homology from Prdm15 were done using the ClustalW and Clustal Omega multiple sequence alignment tools from the EMBL-EBI homepage and QIAGEN CLC Genomics Workbench version 7.7.3 (https://digitali.nsights.qiagen.com/). The following sequences from the NCBI GenBank were used: *Homo sapiens*: NP_071398.5; *Mus musculus*: NP_659038.2; *Xenopus laevis*: XP_018101368.1; *Xenopus tropicalis*: NP_012813515.1; and *Danio rerio*: NP_009303772.1.

### 4.2 *Xenopus laevis* embryos

*X. laevis* embryos were generated, cultured, and staged according to the standard protocols (Nieuwkoop and Faber, 1994; Sive et al., 2010). All experimental procedures were performed in agreement with the German animal use and care law. Furthermore, *in vivo* experiments were approved by the administration of the state of Baden-Württemberg (Regierungspräsidium Tübingen). Embryos were cultivated in 0.1× modified Barth’s saline with HEPES buffer (MBSH) and fixed with 1× MEMFA(T) [0.1 M MOPS, pH 7.4; 2 mM EGTA, 1 mM MgSO4 (H_2_O)7, 4% formaldehyde, 0.1% Tween] at the desired stage.

### 4.3 Morpholino oligonucleotide and microinjections

To perform KD experiments, Prdm15 morpholino oligonucleotide (MO) (5′-TCA​TTC​ACA​CCT​GCT​CCT​CAA​TAG​C-3′) and control MO (CoMO) (5′-CCT​CTT​ACC​TCA​GTT​ACA​ATT​TAT​A-3′) were purchased from Gene Tools (Philomath, OR, United States). The MOs were diluted in diethyl-pyro-carbonate (DEPC)–treated water. The translational blocking efficiency of the used Prdm15 MO was previously described in Mann et al. (2021). To target the anterior neural tissue, 15 ng of Prdm15 MO was injected unilaterally into one dorsal animal blastomere of eight-cell-stage *X. laevis* embryos (Moody and Kline, 1990). The un-injected side served as an internal control. As injection control, control MO (CoMO) was injected (15 ng). Successful and correct injections were controlled by the co-injection of 0.5 ng RNA coding for *GFP*. For rescue experiments with different human *PRDM15* constructs, 15 ng of Prdm15 MO was co-injected with either 0.5 ng human full-length *PRDM15* or human full-length *PRDM15* with the specific point mutations identified in human patients (*hPRDM15-M154K* and *hPRDM15-C844Y*). For further rescue experiments, the following amounts of RNA were used: 50 pg *wnt4* RNA (Maurus et al., 2005), 100 pg *dshΔDEP* and *dshΔDIX* RNA (Miller et al., 1999), and 1 ng *caJNK*1 RNA (Cizelsky et al., 2014). To adjust the amount of RNA or MO per injection, *GFP* RNA and control MO were used, respectively.

### 4.4 Whole-mount *in situ* hybridization and histology

To study the spatiotemporal expression profile during *X. laevis* embryogenesis, whole-mount *in situ* hybridization (WMISH) analysis was performed according to the established protocols (Hemmati-Brivanlou et al., 1990; Lufkin, 2007). *Digoxigenin* (DIG)-labeled antisense RNA probes were generated by *in vitro* transcription using T7, SP6, or T3 RNA polymerase (Roche, Basel, Switzerland). Subsequently, the embryos were stained with BM Purple (Roche, Basel, Switzerland) for up to 14 days for exterior view or NBT/BCIP (Roche, Basel, Switzerland) for up to 14 days for sectioning. BM Purple–stained embryos were bleached with 30% H_2_O_2_. For more detailed tissue analysis, NBT/BCIP-stained or wild-type *X. laevis* embryos were equilibrated in 1 mL gelatin/albumin solution (2.2 g gelatin, 135 g BSA, 90 g sucrose, and 500 mL 1× PBS) overnight at 4°C and embedded in 1 mL gelatin/albumin solution with 75 µL glutaraldehyde (Fluka, Switzerland). Using a vibratome (Vibratome 1500 Classic, The Vibratome Company), we made sections with a thickness of 25 μm according to Guo et al. (2011). We used the following RNA antisense probes as described previously: *alcam* (*activated leukocyte cell adhesion molecule*) (Gessert et al., 2008a; Cizelsky et al., 2014; Seigfried et al., 2017), *prdm15* (*PR-domain zinc finger domain 15*) (Mann et al., 2021), *celf1* (*CUGBP elav-like family member 1*) (Day and Beck, 2011; Rothe et al., 2017), *cryba1* (*crystallin beta A1*) (Day and Beck, 2011), *egr2* (*early growth response 2*) (Cizelsky et al., 2013), *emx1* (*empty spiracles homeobox 1*) (Cizelsky et al., 2013), *foxd3* (*forkhead box D3*) (Gessert et al., 2007; Li et al., 2009), *otx2* (*orthodenticle homeobox 2*) (Lamb and Harland, 1995), *pax6* (*paired box 6*) (Hitchcock et al., 1996; Hollemann et al., 1998), *pou4f1* (*pou class 4 homeobox 1*) (Liu et al., 2000), *prox1* (*prospero homeobox 1*) (Dyer et al., 2003), *rax* (*retina and anterior neural fold homeobox*) (Furukawa et al., 1997), *rgma* (*repulsive guidance molecule a*) (Gessert et al., 2008b), *rho* (*rhodopsin*) (Chang and Harris, 1998), *snai2* (*snail family zinc finger 2*) (clone ID: pMX363), *sox3* (*sex-determining region Y-box 3*) (Maurus et al., 2005), *tubb2b* (*tubulin beta 2B class IIb*) (Moody et al., 1996), *twist1* (*twist family bHLH transcription factor 1*) (Gessert et al., 2007), *vsx1* (*visual system homeobox 1*) (Hayashi et al., 2000), and *wnt4* (*wnt family member 4*) (Maurus et al., 2005).

### 4.5 Cartilage staining by Alcian blue and cranial nerve staining by 3A10 antibody

In order to investigate the craniofacial cartilage and cranial nerves, embryos injected with 15 ng Prdm15 MO and CoMO were fixed at the late tadpole stages.

Alcian blue staining: embryos were stained with Alcian blue as previously described (Gessert et al., 2007), and afterward, the cranial cartilage was dissected and imaged.

3A10 antibody staining: embryos were treated with the monoclonal 3A10 antibody (DSHB, Iowa City) to visualize the cranial nerves (Schuff et al., 2007).

### 4.6 Phospho-histone H3 staining

Proliferative cells of stage 23 embryos were stained for phospho-histone H3 (pH H3) according to the established protocols (Gessert et al., 2007; Cizelsky et al., 2013).

### 4.7 Imaging

Representative embryos or experiments were imaged. Whole *X. laevis* embryos from the exterior view were imaged by using an Olympus MVX10 (fluorescence) or Olympus SZX12 microscope and an Olympus UC50 camera. Vibratome sections were imaged with an Olympus BX60 microscope and an Olympus DP70 or an Olympus DP28 camera. Images were processed with ImageJ2 version 2.9.0 (Rueden et al., 2017) and Affinity Designer 1.10.6.

### 4.8 Quantitative tissue and expression measurements

For all quantitative measurements, one representative experiment was used for unilaterally control MO (CoMO) and Prdm15 MO–injected embryos.

The area of the eye, apex angle of coloboma, head area, head width, and interocular distance were measured using the software ImageJ2 version 2.9.0 (Rueden et al., 2017).

For brain area measurements, the brains of stage 42/43 embryos were dissected and imaged. ImageJ2 (Rueden et al., 2017) was used to measure the area of the brain.

To analyze the area and intensity of *wnt4* and *alcam* gene expression, CoMO- and Prdm15 MO–injected embryos were imaged after WMISH. Using ImageJ2 version 2.9.0 (Rueden et al., 2017), the area marked by the boxes (e.g., Figures 7C, D, G–H, boxes indicated by the purple area) was selected and measured. Simultaneously, the corresponding mean intensity of the *wnt4* and *alcam* expression area was measured. For the area measurements, the values (injected/un-injected side) were then divided and analyzed with GraphPad Prism version 9 and 10.0.0 for macOS (Boston, Massachusetts, USA, www.graphpad.com). For the mean intensity analysis, a reference value for the mean intensity was measured for each embryo analyzed, and then the mean intensity of the measured *wnt4* and *alcam* areas was subtracted from the reference value. The calculated values (injected/un-injected side) were then divided and analyzed using GraphPad Prism version 9 and 10.0.0 for macOS (Boston, Massachusetts, USA, www.graphpad.com).

To analyze the area of gene expression from *pax6*, *rax*, *snai2*, and *foxd3*, CoMO-injected, Prdm15 MO–injected, and Prdm15 MO + wnt4 RNA–injected embryos were imaged after WMISH. Using ImageJ2 version 2.9.0 (Rueden et al., 2017), the area of *pax6*, *rax*, *snai2*, and *foxd3* expression was selected as indicated through the boxes and measured (Figures 3F, 8A–F, boxes indicate the measured purple or gray area). The values (injected/un-injected side) were then calculated and analyzed using GraphPad Prism version 9 and 10.0.0 for macOS (Boston, Massachusetts, USA, www.graphpad.com).

### 4.9 Statistics

Data were analyzed with version 9 and 10.0.0 for macOS (Boston, Massachusetts, USA, www.graphpad.com). Only experiments with a higher survival rate than 50% were considered for statistical evaluation. Statistical evaluation was performed only with more than three independent experiments. To determine statistical differences, the non-parametric Mann–Whitney rank-sum test was used, and the error bars represent the standard errors of the mean (SEM). Statistical significances are indicated as *ns*, *p > 0.05*; *, *p* ≤ 0.05; **, *p* ≤ 0.01; ***, *p* ≤ 0.001; and ****, *p* ≤ 0.0001.

## Data Availability

The original contributions presented in the study are included in the article/Supplementary Material; further inquiries can be directed to the corresponding author.
