# Supplementary material for: Prdm15 acts upstream of Wnt4 signaling in anterior neural development of Xenopus laevis
Source: Front Cell Dev Biol. 2024 Feb 20;12:1316048. doi: 10.3389/fcell.2024.1316048 (PMC10912572; doi:10.3389/fcell.2024.1316048)
Supplement: Supplementary file 1 [file Presentation1.pdf]

Supplementary Material

Prdm15 acts upstream of WNT4 signaling in anterior neural development of *Xenopus laevis*

This Supplementary Material contains further Supplementary Figures 1-5.

A

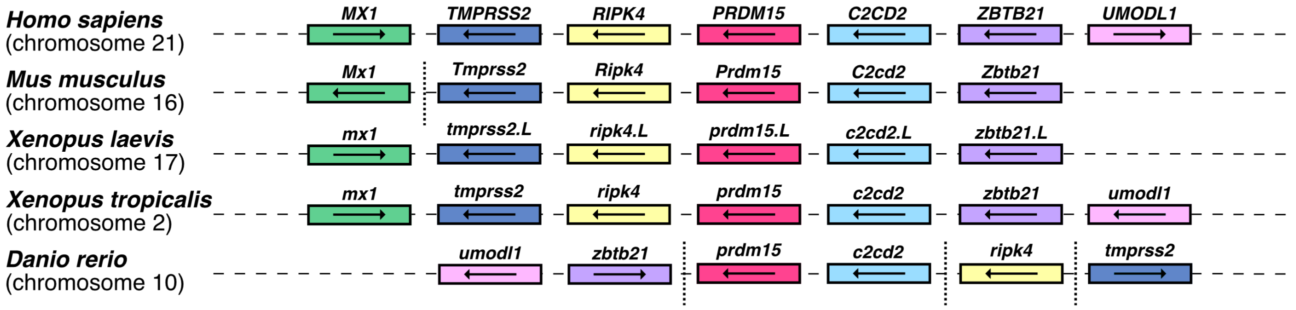

B

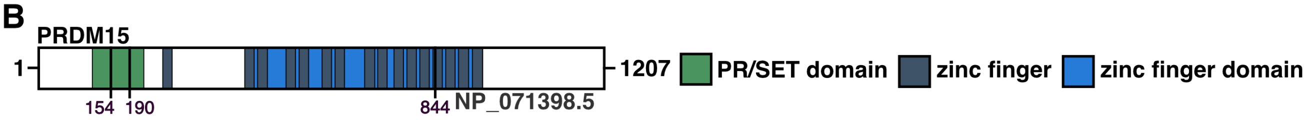

C **NP_071398.5 PR domain zinc finger protein 15 isoform 1 [Homo sapiens]**

1– MAEDGSEEIMFIWCEDCSQYHDSECPELGPVVMVKDSFVLSRARSWPASGHVHTQAGQGMRGYEDRDRADPQQLPEA
VPAGLVRRLSGQQLPCRSTLTWGRLCHLVAQGRSSLPPNLEIRRLEDGAEGVFAITQLVKRTQFGPFESRRVAKWEKESA
FPLKVFQKDGHPVCFDTSNEDDCNW**M**MLVRPAAEAEHQNLTAYQHGSDVYFTTSRDIPP**G**TELRVWYAAFYAKKMDKP
MLKQAGSGVHAAGTPENSAPVESEPSQWACKVCSATFLELQLLNEHLLGHLEQAKSLPPGSQSEAAAPEKEQDTPRGEP
PAVPESENVATKEQKKKPRRGRKPKVSKAEQPLVIVEDKEPTEQVAEIITEVPPDEPVSATPDERIMELVLGKLATTTTDTS
SVPKFTHHQNNTITLKRSLILSSRHGIRRKLIKQLGEHKRVYQCNICSKIFQNSSNLSRHVRSHGDKLFKCEECAKLFSRKES
LKQHVSYKHSRNEVDGEYRYRCGTCEKTFRIESALEFHNCRTDDKTFQCEMCFRFFSTNSNLSKHKKKHGDKKFACEVC
SKMFYRKDVMLDHQRRHLEGVRRVKREDLEAGGENLVRYKKEPSGCPVCGKVFSCRSNMNKHLLTHGDKKYTCEICGR
KFFRVDVLRDHIHVHFKDIALMDDHQREEFIGKIGISSEENDDNSDESADSEPHKYSCKRCQLTFGRGKEYLKHIMEVHKE
KGYGCSICNRRFALKATYHAHMVIHRENLPDPNVQKYIHPCEICGRIFNSIGNLERHKLIHTGVKSHACEQCGKSFARKDML
KEHMRVHDNVREYLCAECGKGMKTKHALRHHMKLHKGIKEYECKECHRRFAQKVNMLKHCKRHTGIKDFMCEL**C**GKTF
SERNTMETHKLIHTVGKQWTCSVCDKKYVTEYMLQKHVQLTHDKVEAQSCQLCGTKVSTRASMSRHMRRKHPEVLAVRI
DDLDHLPETTTIDASSIGIVQPELTLEQEDLAEGKHGKAAKRSHKRKQKPEEEAGAPVPEDATFSEYSEKETEFTGSVGDE
TNSAVQSIQQVVVVTLGDPNVTTPSSSVGLTNITVTPITTAAATQFTNLQPVAVGHLTTPERQLQLDNSILTVTFDTVSGSAML
HNRQNDVQIHPQPEASNPQSVAHFINLTTLVNSITPLGSQLSDQHPLTWRAVPQTDVLPPSQPQAPPQQAAQPQVQAEQ
QQQQMYSY **–1207**

D

species	protein length (aa numbers)	protein homology (%)		
		overall	PR/SET domain	Zinc finger domain
Homo sapiens	1207	100	100	100
Mus musculus	1174	91.66	92.79	97.05
Xenopus laevis	1197	71.21	71.43	88.29
Xenopus tropicalis	1202	71.69	72.97	88.69
Danio rerio	1120	66.07	67.57	82.78

E

	Met154Lys ⬇	Glu190Lys ⬇	Cys844Tyr ⬇
Homo Sapiens	EDDCNWMMLVRPA	PGTELRVWY	KDFMCELCGKTFSE
Mus musculus	EDDCNWMMLVRPA	AGTELRVWY	KDFMCELCGKTFSE
Xenopus laevis	EDDCNWMMFVRPA	LGAELRVWY	KDFMCELCGKTFSE
Xenopus tropicalis	EDDCNWMMFVRPA	LGAELRVWY	KDFMCELCGKTFSE
Danio rerio	EDDCNWMMLVRPA	PGTELRVWY	KDFMCELCGKTFSE

Supplementary Figure 1. *In silico* analysis of *prdm15* revealed a high conservation across species. (A) Synteny analysis of *prdm15* and its neighboring genes from *Homo sapiens*, *Mus musculus*, *Xenopus laevis*, *Xenopus tropicalis* and *Danio rerio*. Schematic overview of the genes` location and its surrounded genes among different species. Conserved genes are depicted by boxes with identical colors. *Prdm15* is shown in red; non-conserved neighboring genes are not shown. The orientation of the open reading frames is indicated by arrows. Gene length as well as distances are not proportional to their actual size. More distanced genes on the same chromosome are emphasized by a vertical dashed line. Chromosomal location is listed below the species name. *X. laevis* L-chromosome was investigated. **(B)** Protein domains of human PRDM15. PR/SET domain (green) and zinc finger domain (light blue) as well as zinc finger motifs (dark blue) are shown. The positions of the mutated protein in the PR/SET (p.M154K (c.461T>A); p.G190K (c.568G>A); Mann et al., 2021; Mzoughi et al., 2020) and zinc finger domain (p.C844Y (c.2531G>A); Mann et al., 2021; Mzoughi et al., 2020) in GAMOS patients are indicated. **(C)** Human PRDM15 protein sequence. Amino acids, the PR/SET and zinc finger domains and the mutated proteins (in bold) are marked **(D)** Homology of the amino acid sequences of full-length PRDM15. Amino acid length is given in numbers. Percentages represent identical residues (percent identity) of the indicated species compared to *Homo sapiens*. In addition, protein homology of the different domains was investigated showing even a higher conservation. **(E)** The alignment across indicated species show that the positions of the mutated genes in the PR/SET (c.461T>A, p.M154K; c.568G>A, p.G190K; Mann et al., 2021; Mzoughi et al., 2020) and zinc finger domain (c.2531G>A, p.C844Y; Mann et al., 2021; Mzoughi et al., 2020) in GAMOS patients are evolutionarily conserved between human, mouse, frog and fish. Abbreviations: aa, amino acid; *c2cd2*, *c2 calcium-dependent domain containing 2*; Cys844Tyr, Cysteine844Tyrosine; Glu190Lys, Glutamine190Lysine; Met154Lys, Methionine154Lysine; *mx1*, *mx dynamin-like GTPase 1*; Prdm15, PR-domain zinc finger protein 15; *ripk4*, *receptor interacting serine/threonine kinase 4*; *tmprss2*, *transmembrane serine protease 2 gene 1*; *umodl1*, *uromodulin like 1*; *zbtb21*, *zinc finger and BTB domain containing 21*.

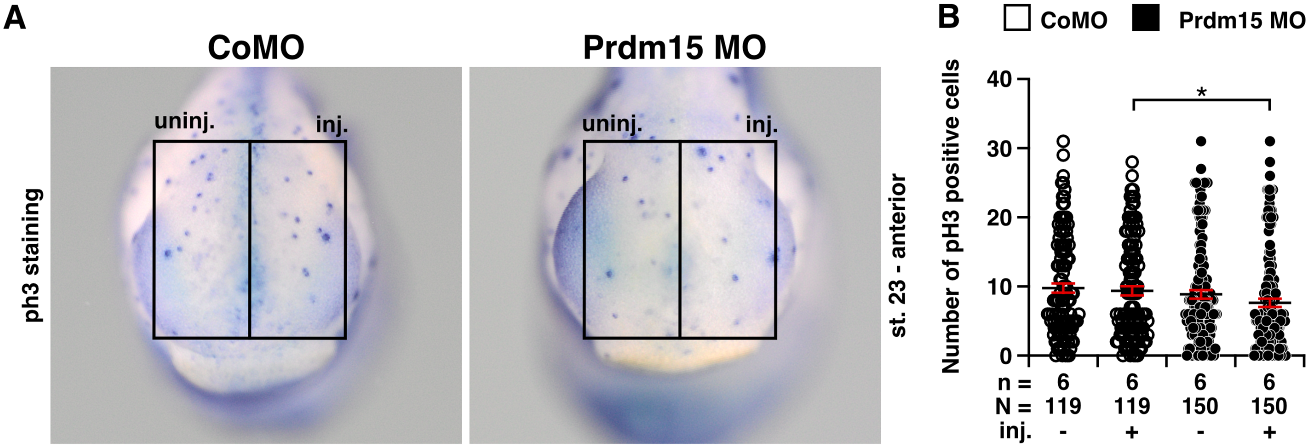

Supplementary Figure 2. Prdm15 knockdown leads to less proliferation. (A) Proliferative cells of embryos injected with Control MO (CoMO) or Prdm15 MO were stained with a phospho-histone H3 (pH H3) antibody at stage 23. The anterior view of embryos was imaged and the number of proliferative cells were counted. The area of the anterior neural plate of the injected side and the un-injected side were compared. The black boxes indicate the area where proliferative cells were counted. **(B)** Statistical evaluation of the number of pH H3 positive cells as illustrated in (A) shows a significant reduction in expression of the number of pH H3 positive cells on the injected side upon Prdm15 knockdown, whereas the un-injected side and CoMO-injected embryos are not affected. Abbreviations: CoMO, control morpholino oligonucleotide; inj., injected; MO, morpholino oligonucleotide; n, number of independent experiments; N, number of analyzed embryos in total; pH H3, phospho-histone H3; Prdm15, PR-domain zinc finger protein 15; uninj., un-injected. Error bars indicate standard errors of the means. **, $p \leq 0.05$.*

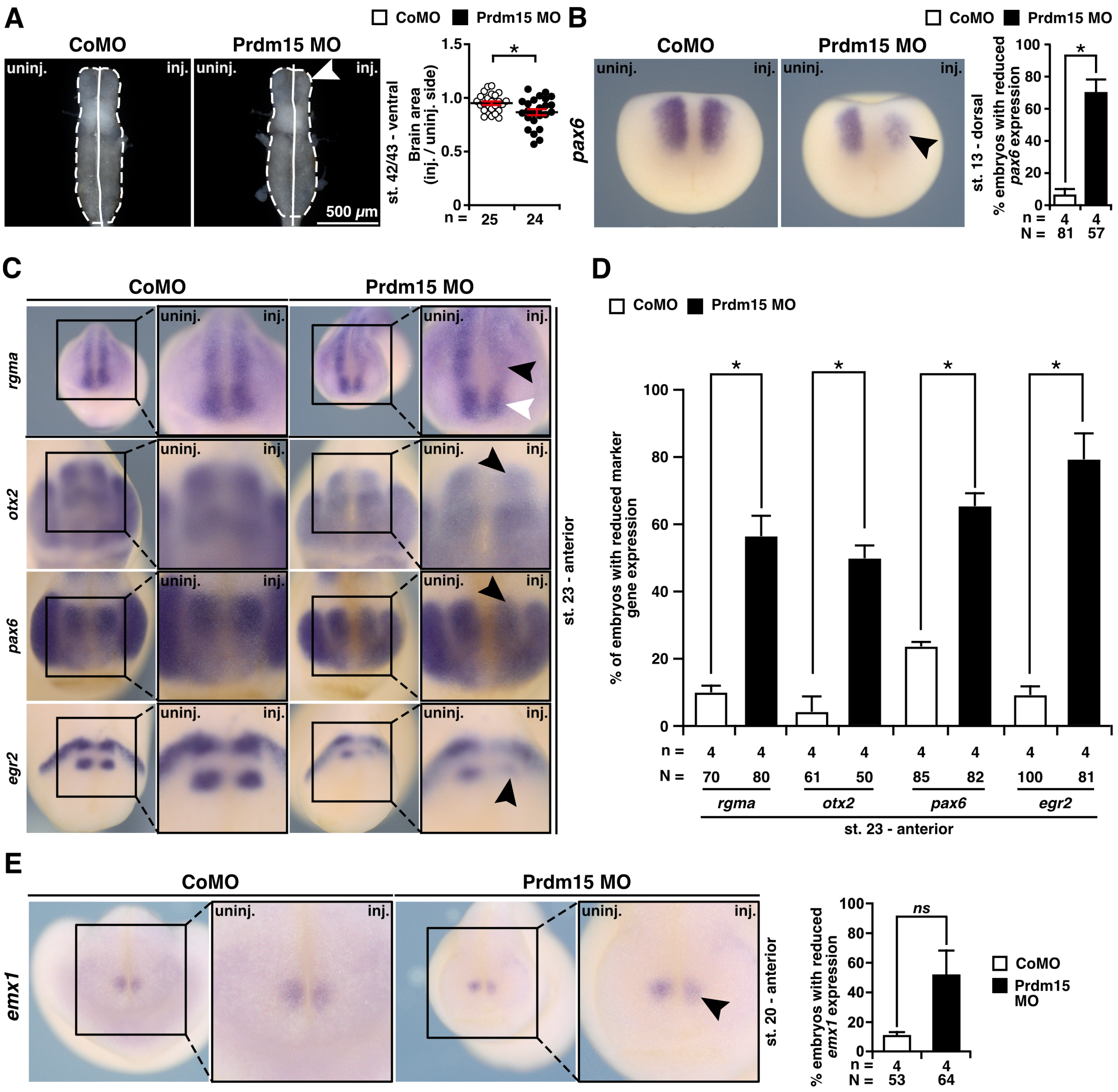

Supplementary Figure 3. Prdm15 MO injection hinders proper development of the brain. (A) Ventral view of isolated brains from control and Prdm15 morphants (stage 42/43). Prdm15 knockdown (KD) resulted in a significant smaller brain area on the injected side (white arrowhead). **(B)** Dorsal view of Control MO (CoMO) and Prdm15 MO-injected embryos showed a reduced expression of *pax6* at stage 13 upon Prdm15 KD (black arrowhead) visualized by whole mount *in situ* hybridization (WMISH). **(C)** Anterior view of CoMO and Prdm15 MO-injected embryos at stage 23 after WMISH with brain specific genes such as *rgma, otx2*, *pax6* and *egr2* revealed a reduced marker gene expression on the Prdm15 MO-injected side in the mid- and hindbrain (black arrowheads) and slightly in the forebrain region (white arrowhead). **(D)** Statistical evaluation of the brain specific gene expression as

illustrated in (C) shows a significant reduction in expression of all marker genes upon Prdm15 MO KD. **(E)** Anterior view of CoMO and Prdm15 MO-injected embryos at stage 20 after WMISH with the forebrain specific marker gene *emx1* revealed a reduced *emx1* expression on the Prdm15 MO-injected side (black arrowheads) for less than half of the embryos. Statistical evaluation shows no significant reduction in *emx1* expression upon Prdm15 MO KD. Abbreviations: CoMO, control morpholino oligonucleotide; *egr2*, *early growth response 2*; inj., injected; *emx1*, *empty spiracles homeobox1*; KD, knockdown; MO, morpholino oligonucleotide; n, number of independent experiments; N, number of analyzed embryos in total; *ns*, non-significant; *otx2*, *orthodenticle homeobox 2*; *pax6*, *paired box 6*; Prdm15, PR-domain zinc finger protein 15; *rgma*, *repulsive guidance molecule a*; uninj., un-injected; WMISH, whole mount *in situ* hybridization. Error bars indicate standard errors of the means. *ns*, $p > 0.05$; *, $p \leq 0.05$.

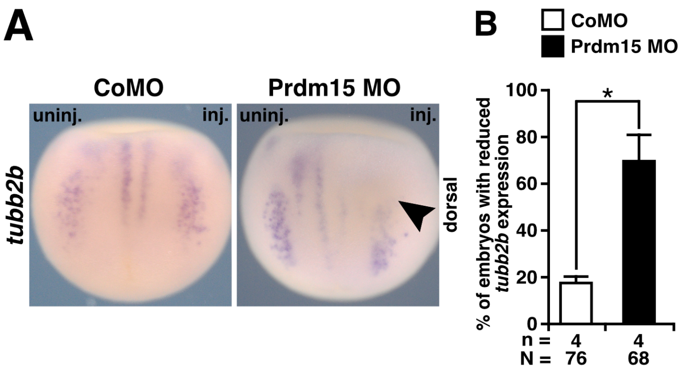

Supplementary Figure 4. Prdm15 knockdown influences *tubb2b* expression. Dorsal view of stage 13 embryos treated with a *tubb2b* probe to perform a whole mount *in situ* hybridization. The expression analysis confirms the reduced gene expression in the dorsal neural tissue (black arrowhead) upon Prdm15 KD. Abbreviations: CoMO, control morpholino oligonucleotide; inj., injected; MO, morpholino oligonucleotide; n, number of independent experiments; N, number of analyzed embryos in total; Prdm15, PR-domain zinc finger protein 15; *tubb2b*, *tubulin beta 2B class IIb*; uninj., un-injected. Error bars indicate standard errors of the means. *, $p \leq 0.05$.

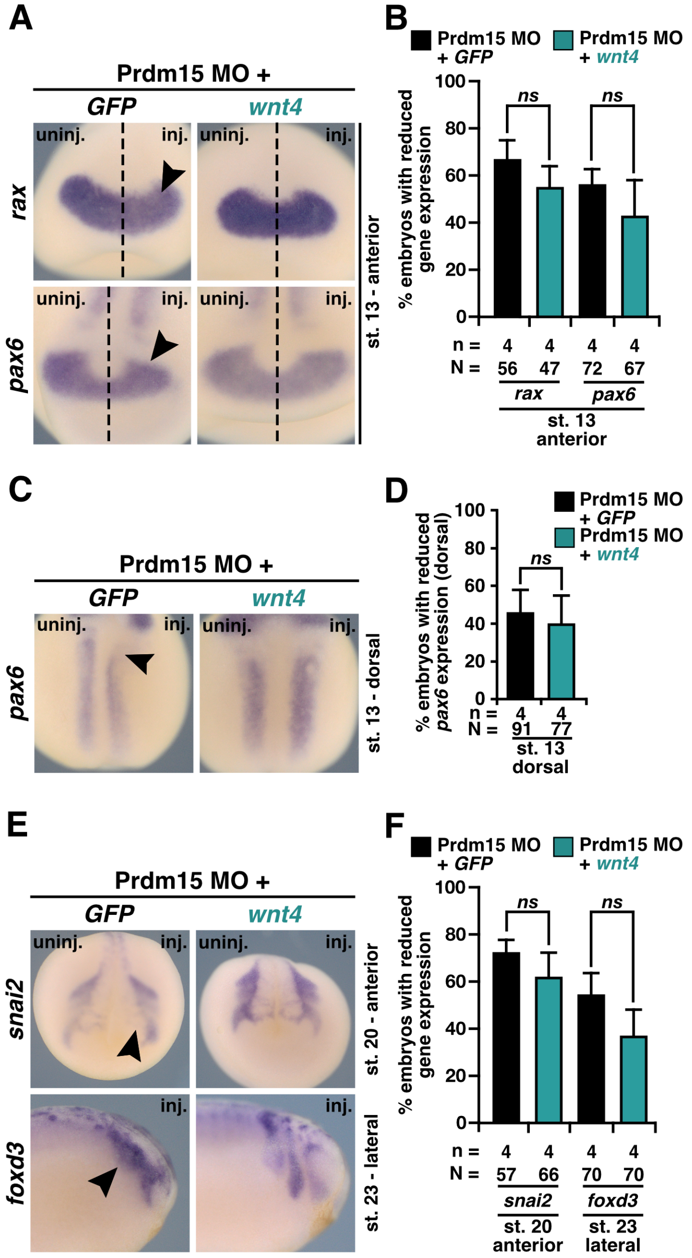

Supplementary Figure 5. Prdm15 MO knockdown (KD) leads to a reduced gene expression that shows a partial rescue by *wnt4* RNA. (A) Anterior view of stage 13 embryos injected with Prdm15 MO in combination with *wnt4* RNA shows the expression of *rax* and *pax6* analyzed with a light microscope. Black arrowheads point to the reduced expression of *rax* and *pax6.* **(B)** Statistical evaluation of *rax* and *pax6* expression as described in (A) reveals that the co-injection of *wnt4* RNA partially rescued the Prdm15-induced reduced marker gene expression. **(C)** Dorsal view of stage 13 embryos injected with Prdm15 MO in combination with *wnt4* RNA shows the expression of *pax6* analyzed with a light microscope. Co-injection of *wnt4* RNA is partially able to rescue the Prdm15-induced reduced *pax6* expression. Black arrowhead points to the reduced expression of *pax6.* **(D)** Statistical

evaluation of *pax6* expression as described in (C). **(E)** Anterior and lateral view of stage 20 respective stage 23 embryos injected with Prdm15 MO in combination with *wnt4* RNA shows the expression of *snai2* and *foxd3* analyzed with a light microscope. Black arrowheads point to the reduced expression of *snai2* and *foxd3.* **(F)** Statistical evaluation of *snai2* and *foxd3* expression as described in (E) reveals a partial rescue of the Prdm15-induced reduced marker gene expression. Abbreviations: *foxd3*, *forkhead box D3*; *GFP*, green fluorescent protein; inj., injected; MO, Morpholino Oligonucleotide; n, number of independent experiments; N, number of analyzed embryos in total; *ns*, non-significant; *pax6*, *paired box 6*; Prdm15, PR-domain zinc finger protein 15; *rax*, *retina and anterior neural fold homeobox*; *snai2*, *snail family transcriptional repressor 2*; st., stage; uninj., un-injected; *wnt4*, *wnt family member 4*. Error bars indicate standard errors of the means. *ns*, $p > 0.05$.
